# Visualizing health inequality data: guidance for selecting and designing graphs and maps

**DOI:** 10.1186/s12939-025-02667-0

**Published:** 2025-12-11

**Authors:** Nicole Bergen, Katherine Kirkby, Devaki Nambiar, Anne Schlotheuber, Ahmad Reza Hosseinpoor

**Affiliations:** Department of Data, Digital Health, Analytics and AI, World Health Organization, 20 Avenue Appia, Geneva 27, CH-1211 Switzerland

**Keywords:** Data visuals, Graph, Health equity, Health inequality, Knowledge translation, Map, Reporting, Visualization

## Abstract

Several resources and tools have been developed for carrying out health inequality analysis, including the preparation of disaggregated data and calculation of summary measures of health inequality. There is also a body of literature that addresses best practices for the preparation of commonly used graphs and maps. There is, however, little guidance for the selection and design of appropriate data visuals for corresponding health inequality data and assessments. This paper aims to fill this gap by describing the types of graphs and maps most frequently used for reporting health inequality analyses and recommending how they can be effectively applied to distinct reporting scenarios. For a range of different data visual types, we assess how they can be designed for effectiveness, attending to the principles of accuracy, utility and efficiency. We assess the suitability of different data visuals for reporting the latest status of inequality, change in inequality over time, inequality assessments across settings and impact of eliminating inequality, considering how different data visualization approaches may be needed for reporting disaggregated data versus summary measures of inequality. Effective visuals can enhance the impact of health inequality reporting and support evidence-informed action to advance health equity.

**Supplementary Information:**

The online version contains supplementary material available at 10.1186/s12939-025-02667-0.

## Introduction

Health inequality monitoring is central to evidence-informed efforts to advance health equity [1, 2]. Health inequalities are defined as measured differences in health between population subgroups that have customarily referred to health differences associated with social advantage and disadvantage. They provide insight into where policies, programmes and practices can be focussed to address situations that are unfair, avoidable and remediable (in other words, inequitable) [3]. While a plethora of tools and resources has been developed to characterize health inequality data analysis techniques and make them more approachable [3–6], guidance specific to the selection and preparation of data visuals for health inequality analyses remains limited [7]. One tool that helps to fill this gap is the WHO Health Equity Assessment Toolkit (HEAT), a software application that facilitates the analysis of health inequality data and generates four types of data visuals [8].

Data visualization refers to “the graphical representation of information and data, using visual elements like charts, graphs, and maps to provide an accessible way to understand trends, outliers, and patterns in data” [9]. Well-designed visuals can strengthen the presentation of health data and bolster its impact as part of evidence-informed responses [10, 11]. Data visuals, such as graphs and maps, can make patterns in the data apparent, aid interpretation and help readers draw conclusions. Often presented in a “static” format, graphs and maps may also be part of interactive data visuals, allowing the reader to navigate, select and customize how they view large datasets [3].

The topic of effective data visual design has been explored by fields as diverse as psychology, education, health communication, political science, statistical cognition, computer and information sciences, and journalism [12]. Thus, different priorities are apparent when answering the question of “what constitutes an effective visual?” [13]. For instance, some prioritize a data-centric approach, emphasizing the accuracy with which the attributes of the visual match the attributes of the data [14], and the efficient use of visual elements to represent the data [10]. Accordingly, some have suggested that larger and more complex datasets require more sophisticated visuals [15]. Others have prioritized a task-centric view, with the design of a visual aligned with a particular outcome [16], or the ease of interpretation [17].

Previous literature about visualizing health inequality data has tended to adopt a narrow scope of focus on a particular inequality analysis approach or a few types of visuals. For example, Blakely et al. (2017) developed a typology for presenting time trends in average mortality alongside changes in absolute or relative inequality using arrow charts [18]. Asada et al. (2017) contrasted the use of bar graphs and dot charts, advocating for increased use of the former in health inequality data presentation [19]. Studying the suitability of different graph types for making comparisons between groups, Zhao and Gaschler (2022) compared the speed and ease of interpreting bar, line and pie graphs, concluding that bar graphs yielded the fastest comparisons [20]. Kjellsson and Petrie (2017) considered how the design of health inequality graphs can account for the underlying value judgements of inequality measures [21]. Exploring ways to visualize absolute inequality, the International Centre for Equity in Health proposed the equiplot (a type of dot plot) as a graph with specific applications to health inequality reporting [22, 23].

The selection and design of data visuals impact how they are interpreted, as well as subsequent decisions that may be taken based on the data [24]. Those involved in preparing and presenting health inequality evidence can benefit from updated and consolidated guidance for how to effectively present health inequality data using data visuals. The first objective of this article is to describe the common types of graphs and maps in health inequality reporting and discuss how they can be used effectively. The second objective is to make recommendations for the selection and design of graphs and maps for particular aspects of health inequality reporting. We describe four scenarios for reporting on the state of inequality (that is, reporting the latest status of inequality, change in inequality over time, assessing inequality across settings and impact of eliminating inequality) and propose effective types of visuals for presenting the corresponding results.

## Approach

To meet our first objective, we scanned health inequality reports and literature to identify common types of data visuals used in health inequality reporting. This included materials available on the World Health Organization (WHO) Health Inequality Monitor, such as the *State of inequality* and *Explorations of inequality* report series, the data visualization components of HEAT and peer-reviewed publications (which were prepared in collaboration with experts from diverse backgrounds) [25]. This yielded a base list of 14 types of visuals: arrow chart, bar graph, box plot, bullet graph, choropleth map, dot plot/equiplot, heatmap, line graph, Sankey diagram, scatterplot, stacked bar graph, strip plot, violin plot and concentration curve. To verify this list, we reviewed all articles published in the International Journal for Equity in Health (IJEqH) over the previous year that included at least one data visual (Box 1).

**Box 1:** Search protocol

The purpose of the search was to identify the types of graphs and maps used to present health inequality data, and to describe the frequency of their use in recent research articles. We conducted our search of the International Journal for Equity in Health because, per the journal aims, articles often report on situations of health inequality using graphs and maps. Because the journal has an international and interdisciplinary focus, the articles capture diverse research outputs. The search included
all articles published over a one-year period, between May 1, 2024 and May 1, 2025. We scanned all articles published online during that period and retained those with at least one graph or map that presented health inequality data. The types of graphs and maps were recorded. Infographics and graphs or maps showing information not related to health inequality data were excluded.

Drawing from Zhu (2007), we discuss the use of each data visual type for reporting health inequality data with respect to three principles of effectiveness: accuracy, utility and efficiency [13]. Accuracy describes visual elements matching the attributes of data elements, such as the presentation of data using ranges versus exact values, and the adoption of appropriate axis scaling. Utility addresses the ability of visuals to help readers achieve a specific task, such as making comparisons between subgroups or assessing changes over time. Efficiency pertains to reducing the cognitive load for a specific task in relation to non-visual representation of the data; that is, the visual should add enhance data interpretation. In addition to these three principles, we also consider factors of prevailing conventions, data literacy and cultural settings, as applicable.

Building on the first objective, our second objective of providing recommendations entailed characterizing four distinct reporting scenarios. These are derived from the components of comprehensive reporting on the state of health inequality in a given health topic, and include data pertaining to (a) the latest status of inequality, (b) change over time, (c) assessing inequality across settings (such as countries or subnational regions) and (d) impact of eliminating inequality [3]. These four scenarios reflect foundational aspects of health inequality monitoring that have wide applicability across reporting outputs. For each scenario, recommendations are accompanied by illustrative examples from published sources. These recommendations are meant to be illustrative of data visual types that are likely to be effective for a given general scenario. The suggested visuals are indicative, meaning that there are other options and possibilities for visualization that may be tailored to surface context-relevant nuance and specificities. It was outside the scope of the article to make recommendations for advanced analysis techniques.

The assessments and recommendations in this paper are based, in part, on the experiences of the author team working on health inequality monitoring over the past two decades. We also relied on relevant peer-reviewed and grey literature and we consulted experts in the areas of data visualization and health inequality assessment. We endeavoured to reflect the most contemporary innovations and developments in the field, as well as established conventions that remain in practice in visualizing health inequality data. The guidance and recommendations in this article are accompanied by justifications and contextualization to encourage readers to make informed choices to enhance the impact of data visuals.

## Data visuals for reporting health inequality data

Our search of IJEqH covered 307 articles published within the previous year, of which 84 contained graphs or maps presenting health inequality data. The findings of this search are summarized in Table 1.

**Table 1:** Types of graphs and maps used to report health inequality data in articles published in the International Journal for Equity in Health (May 1, 2024 to May 1, 2025)

Graph or map type	Frequency of use
Line graph	49
Bar graph	35
Dot plot	32
Choropleth map	23
Lorenz curve/concentration curve	17
Stacked bar graph	16
Scatterplot	14
Equiplot	12
Box plot	3
Heatmap	3
Others: 3D map, combined bar and line graph, density plot, dot density map, drapery plot, hexbin map, isochrone map, path diagram, radar chart, slope index of inequality regression plot, Venn diagram	16 *(use of each graph or map type was limited to 1 or 2 articles)*

**Fig. 1:**
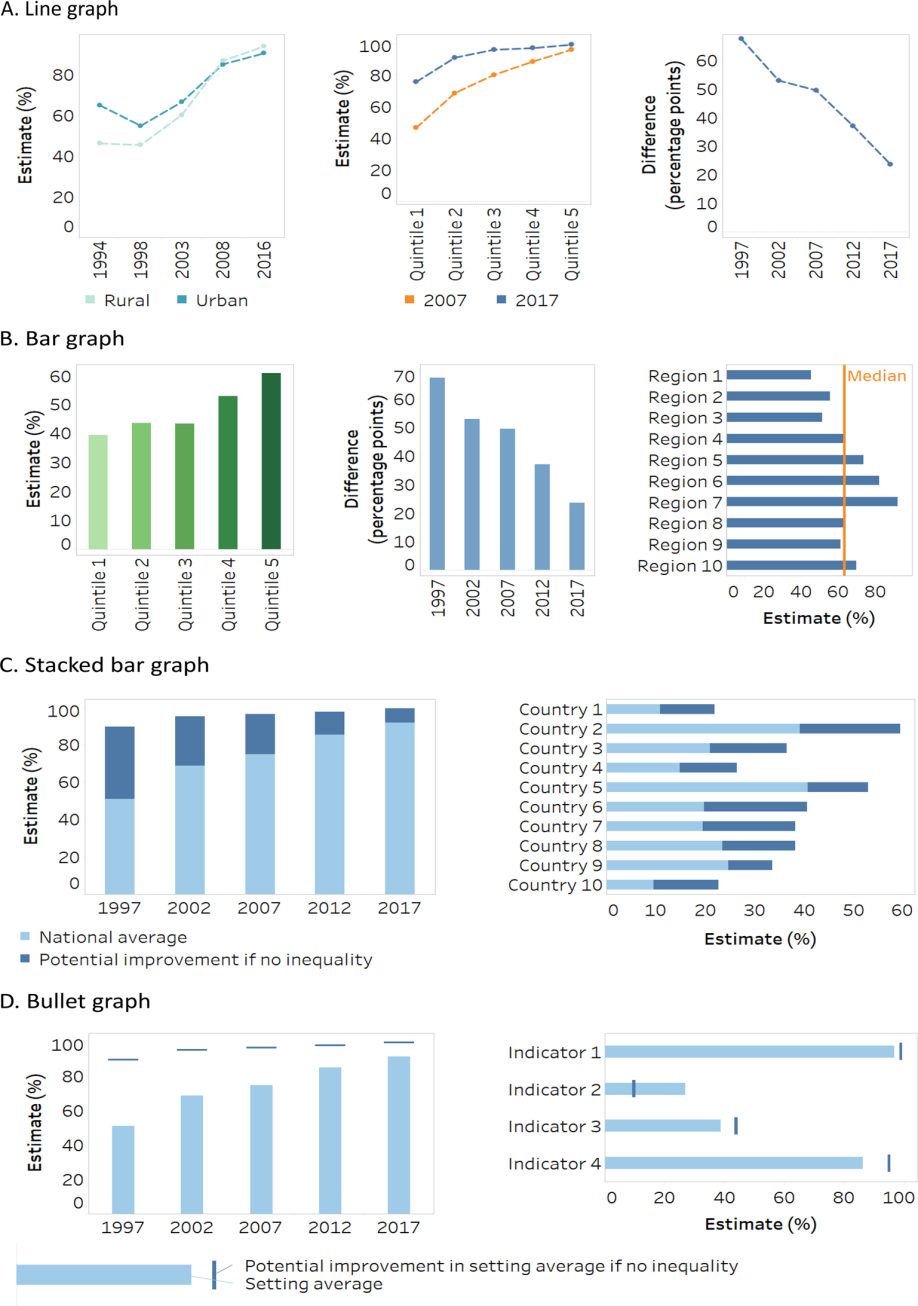

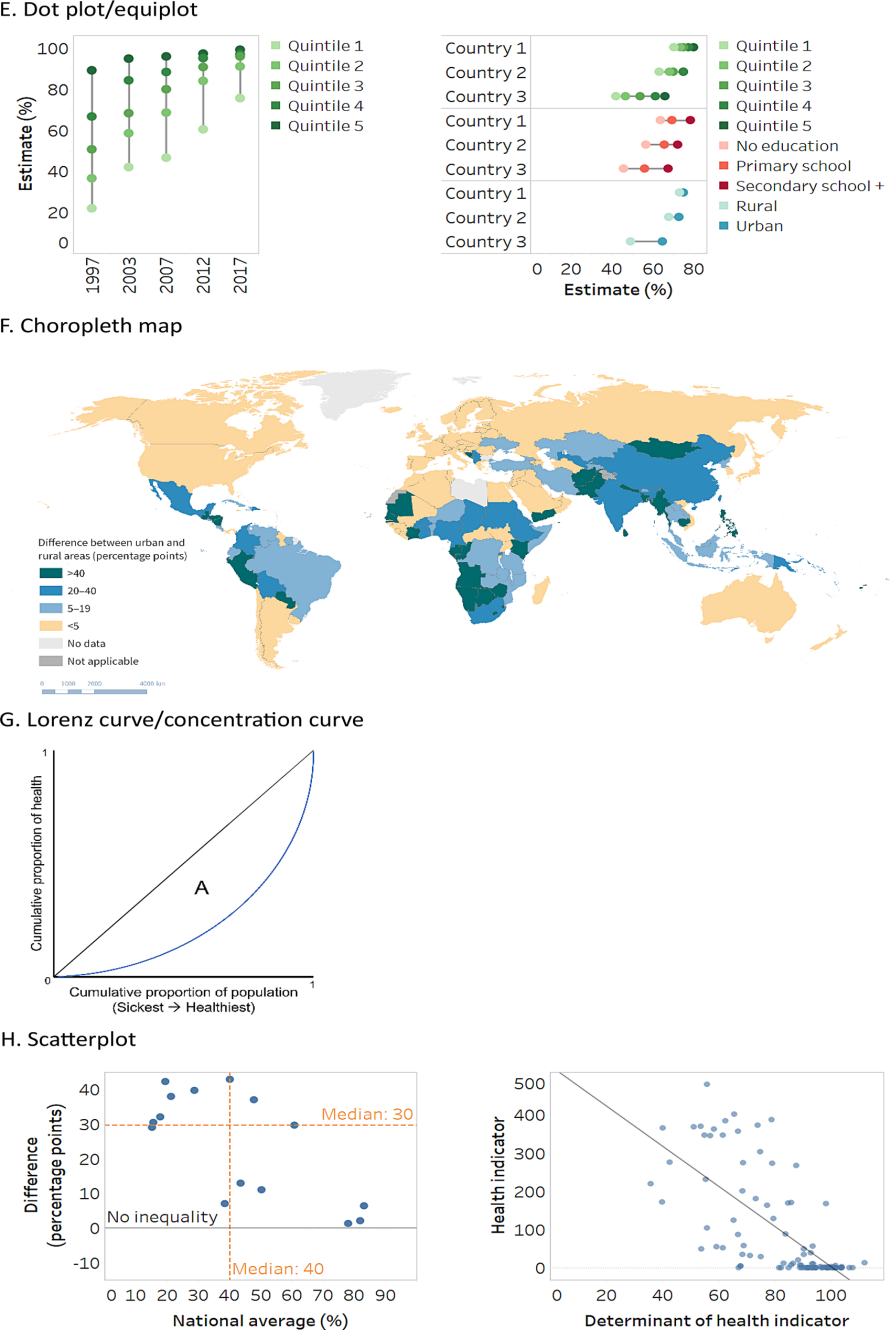

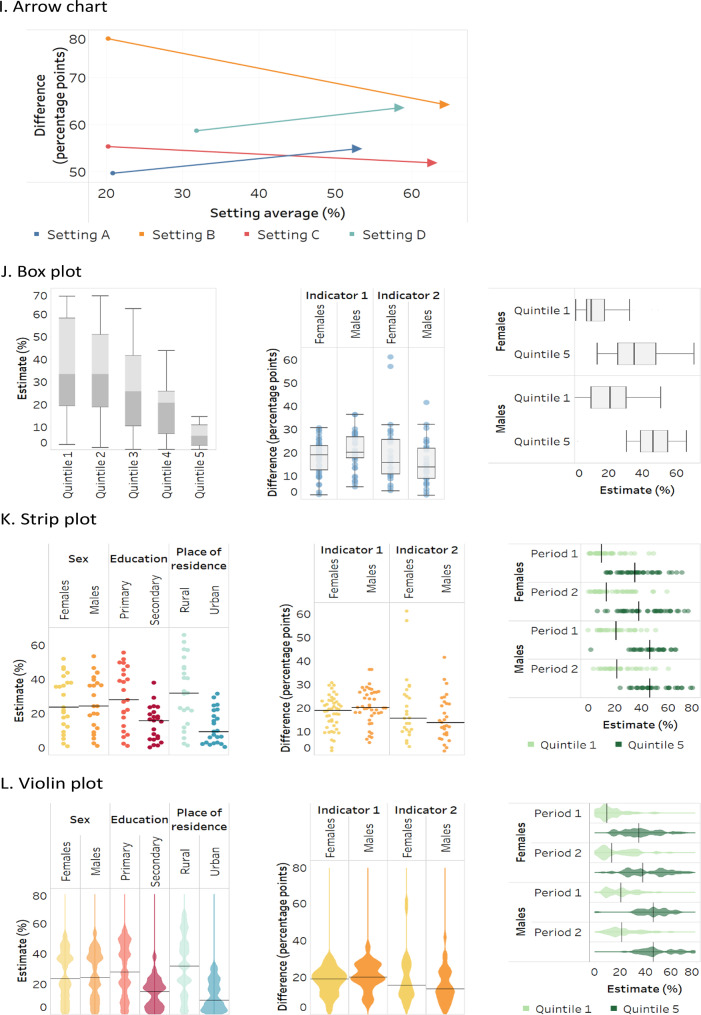

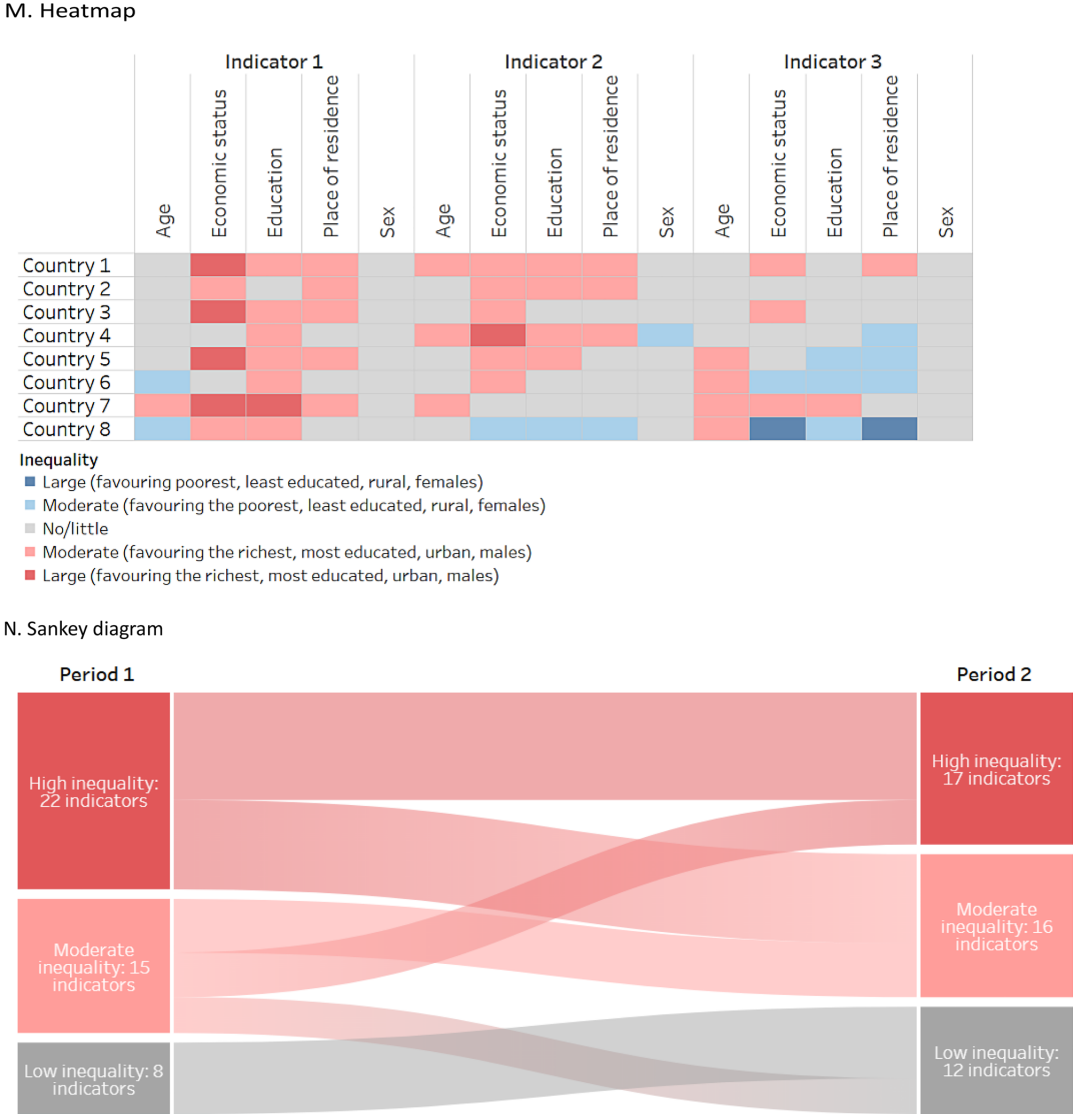
Generic illustrations of common data visuals used in health inequality reporting. Adapted from: World Health Organization [3]

Because stacked bar graphs and bullet graphs share similar attributes and are variations of bar graphs, the three are discussed together. Likewise, due to their similarities, strip plots and violin plots are discussed together, and arrow charts are covered as a variation of a scatterplot. For reference, a generic version of each graph and map type is provided in Fig. 1.

### Line graphs

Line graphs display patterns in data using connecting lines between discrete data points (Fig. 1A). The design or labelling of the lines may denote the subgroup (in the case of disaggregated data) or dimension of inequality (in the case of summary measures of health inequality). Line graphs are often used to show trends over time, displaying a timescale on the horizontal axis and an interval or ratio scale on the vertical axis. In this case, consistent spacing of the timescale should be used.

The slope of the line indicates whether the variable is increasing or decreasing and helps readers to compare the trend in one line against the others. Readers are more likely to describe the data in a line graph as a trend and are less likely to make discrete comparisons between single data points along the line [20, 26]. One source of misinterpretation of line graphs occurs when the trend across discrete data points is described as continuous [26]. That it, the situation between two discrete data points may not match what is indicated by the line connecting those data points.

Confidence intervals can be depicted on a line graph using a shaded range/area. Narrow shading indicates high confidence/low variability. This reminds the audience that the line is an estimate and helps to assess whether differences between lines are statistically meaningful. This is important because inequality data often come from surveys and therefore are estimates.

### Bar graphs

Bar graphs display data using horizontally or vertically oriented bars (Fig. 1B). Information is encoded in the length of the bar and its position along the corresponding axis scale [27]. Bar length is also assessed in relation to other bars. The addition of data labels enhances accuracy of interpreting point values, though may detract from the readability of the graph. Placing numbers inside of bars (rather than next to the bars) has been suggested as a measure to declutter bar graphs [17].

Horizontal bar graphs tend to be well suited for presenting disaggregated data for non-ordered subgroups, such as subnational regions or ethnic groupings [28]. The horizontal orientation facilitates more legible labels (e.g. if subgroup labels are long) as they can be right aligned along the vertical axis. Vertical bar graphs, sometimes termed column graphs, are well suited for ordered subgroups, such as wealth quintiles or education groupings.

Bar graphs are ideal for making comparisons between groups [20]. Sorting the bars in a bar graph can add insight (e.g. placing bars in ascending or descending order) or facilitate interpretation (e.g. sorting from the least to most advantaged subgroup). A line can be added across bars to indicate a value of interest, such as an overall average or a benchmark value. Confidence intervals can be depicted via error bars (lines with/without caps extended above and below each bar) to communicate uncertainty around the estimates.

Bar graphs are a familiar data visual and tend to be intuitive for readers to interpret. They are a default data visual type widely available in data analysis and visualization software, including HEAT [8]. Bar graphs, however, may be less precise visuals compared with those with similar use cases (such as dot plots and equiplots [19]) because the reader is required to decode information about the bar length/area as well as its position along the axis scale. Additionally, bar graphs are subject to a so-called “within the bar bias” – whereby, for bars displaying means, data points are erroneously perceived to fall inside (as opposed to outside) the bar, as if the bar “contains” the relevant data [29].

A stacked bar graph is a variation of a bar graph, where different sections of the bar denote components of the total bar length (Fig. 1C). A bullet graph is a bar graph that uses both bars and lines, with a thin line intersecting or appearing above each bar, often providing information about a benchmark or target value (Fig. 1D). In inequality analysis, these types of graphs are suited for presenting potential increases or decreases in the values of an indicator. Another variation of a bar graph is a histogram, which conveys additional information (e.g. subgroup population size) via the width of the bars.

### Dot plots/equiplots

Dot plots, sometimes called dot charts, and equiplots present information using a single symbol (often a dot) per data point, placed along an axis (Fig. 1E). They may be oriented horizontally or vertically. For a horizontally oriented plot, the horizontal axis typically shows a scale for numerical values, while the vertical axis provides labels associated with the values [19, 30]. Dot plots are interpreted by evaluating the position of the symbol along a common scale, which facilitates accurate interpretation of the data [27]. They offer flexibility in their design, for example, allowing for the addition of data labels or bars of uncertainty around data points [19], though these may detract from the readability of the graph.

Developed for its application to health inequality data, an equiplot can show disaggregated data for all subgroups of a given dimension of inequality, time point and setting on one line (if horizontal) or row (if vertical). The distance between the dots reflects the magnitude of the inequality between subgroups, and a solid line can be used to connect the two extreme data points, denoting absolute inequality. Subgroups can be colour coded and presented together on one line or column. This permits the inclusion of multiple health indicators and/or dimensions of inequality in one visual. In the case of ordered subgroups, such as wealth quintiles or education groupings, equiplots can effectively display patterns in the data, such as gradients, marginal exclusion, or mass deprivation [3]. Equiplots may be less useful for displaying data consisting of numerous subgroups (e.g. subnational regions or districts), as data points may overlap and be difficult to distinguish. Online equiplot creator tools are available through HEAT [8] and the International Center for Equity in Health, which also provides Stata codes for creating equiplots [22].

### Choropleth maps

Choropleth maps present data by shading or colouring predefined geographical areas (such as states, provinces, census tract areas) in relation to the data value (Fig. 1F). Data values are encoded using a range of values in a corresponding legend – and thus data are presented with a low level of precision. The bounds of these ranges can have substantial impacts on the interpretation of the data [27].

Choropleth maps are suited to make comparisons between areas, or to assess trends across areas. Maps are most effectively used if there are compelling patterns across geographical regions (e.g. data values are higher or lower in a particular cluster of co-located areas, or outliers are apparent). Data that lack a clear geographical pattern are often not effectively presented using maps, especially if there are numerous geographical areas. For example, a study of various maps showing COVID-19 cases across the United States of America found that maps displaying state-level data were more effective in terms of knowledge uptake than maps showing county-level data [31].

Choropleth maps should be used with the caveat that the size of an area on the map does not correspond to the population size or density, and they should note where data are not available. Contested borders or areas should also be noted. One limitation of maps is that they typically only present values and not confidence intervals (although there are ways to show these by using multiple maps).

Choropleth maps are one of the data visual options available in HEAT to showcase data disaggregated by subnational region [8].

### Lorenz curve/concentration curve

A Lorenz curve or concentration curve is a specialized type of graph that presents data about the distribution of a variable in a population (Fig. 1G). When used to report on health inequality, Lorenz curves plot the cumulative proportion of the health indicator against the cumulative proportion of the population, ranked from the sickest on the left to the healthiest on the right [3]. If all subgroups (or individuals) have the same health (i.e. the cumulative proportion of the subgroup or population is exactly matched to the cumulative proportion of health), the Lorenz curve runs along the 45-degree line – sometimes termed the “line of equality”. If there is variation in how health is distributed across the population, the Lorenz curve lies below the line of equality, and the greater the variation, the further the curve lies from the line of equality. Gini index, which, when applied as a measure of health inequality, captures the dispersion of a health indicator across all individuals in a given population, can be visualized using a Lorenz curve [3].

A concentration curve can be used to illustrate the extent to which health is concentrated according to a specified social category, such as education or economic status. Thereby, the cumulative proportion of the population ranked by the ordered social category (the cumulative population share) is plotted on the *x*-axis and the cumulative proportion of the health indicator (the cumulative health share) is plotted on the *y*-axis. The resulting concentration curve is interpreted analogously to the Lorenz curve, although the concentration curve may fall above or below the line of equality. Concentration curves can be used to visualize concentration indices, which express the burden or excess level of a health indicator in population subgroups relative to a reference equal distribution across the population, calculated based on the area between the concentration curve and the line of equality [3].

The interpretation of Lorenz curves and concentration curves relies on the visual inspection of the curved line and its relative position to the line of equality. While these visuals can be illustrative of the calculation approach, the extent of inequality may not be evident from the curved line on the graph, and it is not possible to interpret intersecting curves [32]. The index value may need to be displayed alongside the Lorenz curve/concentration curve to aid interpretation.

### Scatterplots

Scatterplots contain information about two variables, with one variable plotted on each axis (Fig. 1H). They help to visualize patterns that can be useful to identify clusters and outliers, as well as to observe patterns and make comparisons across data points.

One application of scatterplots in health inequality reporting is to present data points across four quadrants. This allows for the data points to be displayed according to the most-desirable quadrant, least-desirable quadrant and the two other “mixed” quadrants. For example, a scatterplot might plot national level change in inequality versus change in national average, making evident the countries in the most desirable quadrant (e.g. narrowing inequality alongside improved national average) and the countries in the least desirable quadrant (e.g. widening inequality and worsening national average). Labels can be added to all data points (or selected data points) to provide more detail or illustrate key messages.

Another common application of scatterplots in health inequality reporting is to illustrate associations between two variables. For example, a summary measure of inequality in a health indicator may be plotted against the national average. If the points in a scatterplot trend a certain way (e.g. upward to the right), it suggests a relationship between them, which may be depicted using a regression line. If the points are scattered with no trend, it suggests that there is no relationship between the variables. Scatterplots may also be used to show the strength of association between social determinants of health and health indicators. An arrow chart may be used as a variation of this application to present information about two time points (Fig. 1I). The blunt end of the arrow denotes an earlier point in time, and the arrowhead denotes a later point in time.

The effectiveness of scatterplots may be hindered by datasets with a large number of items, which may lead to the overplotting and overlapping of data points, making it difficult for readers to extract information [33].

### Box plots

Also called box-and-whisker plots, box plots show the distribution of data points using boxes and lines (Fig. 1J). The top and bottom lines indicate minimum and maximum values, the central line indicates the median^1^ (middle point of values) and the boxes indicate the interquartile range (central 50% of values). Box plots can be used with datasets containing a minimum of five data points, though they can accommodate large datasets. Box plots typically do not contain information about individual data points, though outlier data points (as determined by established criteria) may be plotted outside the box plot [34]. While data points can be displayed in the background of box plots (see strip plots), this adds to the cognitive load of interpretation.

Applied to health inequality reporting, box plots enable quick side-by-side comparisons between the distribution of disaggregated data values, summary measure values, or national averages. In such cases, box plots within a data graphic must each represent the same number of data points, unless additional features are introduced to denote the sample size, such as bar width [34]. The “whiskers” in a box plot should be distinguished from standard error bars or standard deviation bars, which may use similar graphic symbols.

### Strip plots and violin plots

Strip plots and violin plots are variations of box plots, providing more detailed information about the density of the underlying data points [35]. For both strip plots and violin plots, data are organized in columns (or rows), with each column (or row) being a population subgroup in the case of disaggregated data, or a dimension of inequality, health indicator or time point, in the case of summary measures. These plots can also be used to show national averages. A line can be added across the plot to indicate the median value of the dataset.

Strip plots, sometimes called jitter plots, show the distribution of individual data points within a row or column (Fig. 1K). Each data point represents a value in one setting. The data points are offset slightly to avoid overlapping, thereby illustrating density and clustering.

Building on the design of strip plots, violin plots use curved outlines and shading to represent the density of the data points at different values (Fig. 1L). Violin plots can be overlaid to compare inequality across indicators, time points and settings.

### Heatmaps

Heatmaps are a colour coded matrix that portray approximate data values according to specified ranges, encoded by colour (Fig. 1M). Formatted similar to a table, with rows and columns labelled, they may present only colours, or they may also include the data values inside the cells. The use of colour enables the efficient interpretation of patterns in the data compared with the use of numbers.

Heatmaps may adopt thresholds – and corresponding colours – to convey different conclusions about the data. For example, a heatmap of summary measures may use colour coding to show instances of high, medium and low inequality. This can support rapid approximate comparisons of inequality across cells, though sound consideration and justification should be provided for the established thresholds. Further, the interpretation of heatmaps should include the caveat that differences that appear substantial according to the defined thresholds may have limited significance for equity or public health.

Heatmaps usually adopt either sequential or diverging colour schemes [3]. Sequential scales (e.g. dark to light) are appropriate when the data values have no defined middle point, and they tend to be easily interpreted. Diverging colour schemes (e.g. spanning one colour to another) are appropriate when there is a meaningful middle point and values at opposing sides of the middle are to be emphasized (e.g. when there is a directionality of inequality). Red-green continuums should be avoided, as this may pose difficulties for people who have variations in how they perceive colour.

### Sankey diagrams

Sankey diagrams display flows or changes of a set of values from one state to another, or one time point to another (Fig. 1N). They consist of nodes (categories representing groups of data), which are connected via links across two or more stages (often time points). Links are represented using arrows or arcs whose width is proportional to the size of the flow. The size of the nodes reflects the number of corresponding data points. For example, Sankey diagrams may used in health inequality reporting to illustrate the number of countries with different thresholds of inequality (e.g. high, medium and low inequality) across multiple time points.

Sankey diagrams are most useful for expressing broad trends and changes. It may be difficult to trace an individual data point in a Sankey diagram (unless the diagram is interactive). The interpretation of a Sankey diagram is clearer when the number of time points (or stages) is limited. They are considered easy to read and are able to present information in a more intuitive manner than a table, though readers may require contextual information when the diagram is accessed for the first time [36].

## Scenarios for reporting health inequalities

For the reporting scenarios 1, 2 and 3, which address the latest situation of inequality, change over time and assessments of inequality across settings, respectively, it is relevant to distinguish between visuals presenting disaggregated data (which pertain to population subgroups) and summary measures of inequality (which express extent of inequality across population subgroups using a single measure). Disaggregated data allow readers to assess the values for each subgroup and make mental comparisons between them to identify patterns in the data. One limitation that can arise when visualizing disaggregated data is that it can create a “hidden picture” requiring the reader to mentally extrapolate information that is not clearly shown in the visual, for example, to determine the differences between subgroups [28].

Visuals of summary measures reduce the need for the reader to make comparisons between disaggregated data points, though they lose some of the inherent simplicity and detail of disaggregated data visuals. The presentation of summary measures for different dimensions of inequality should be done with caution and necessary caveats for interpretation, as variable numbers of underlying subgroups can complicate the comparability of certain measures [37].

### Scenario 1: latest status of inequality in a single setting

The latest status of inequality in a single setting aims to demonstrate the extent of inequality for a given health topic using the most current available data. This is the most common and straightforward scenario for reporting health inequality analysis and the minimum requirement for reporting.

#### Disaggregated data

Recommended visuals for presenting disaggregated data include equiplots and bar graphs (which have similar use cases) and choropleth maps (which are suggested for data disaggregated by a geographic dimension). Equiplots are recommended for most dimensions of inequality, though are most effective when the dimension of inequality consists of relatively few subgroups and when values are not overlapping. Bar graphs can be effectively used to present disaggregated data for any dimension of inequality, showing one subgroup per bar. For both equiplots and bar graphs, multiple health indicators or dimensions of inequality can be presented within one visual. If confidence intervals are important for the interpretation of estimates, these can be included in bar graphs (but are not ideal to include in equiplots).

Choropleth maps may be appropriate in limited applications in this scenario. A common (and effective) use of maps is to show data disaggregated by subnational regions. For example, an article reporting on subnational regional inequality in access to improved drinking water and sanitation in Indonesia used choropleth maps to facilitate comparisons of coverage across 34 regions (Supplementary material 1, Figure A1) [38]. The maps use a five-step sequential colour scale to illustrate coverage according to defined coverage thresholds. While multiple maps could be prepared to each present disaggregated data points for non-geographical dimensions of inequality, the simultaneous interpretation of findings from multiple maps tends to be onerous to the reader. Therefore, this should be limited to instances where the geographical patterning – and its interpretation – are very apparent.

#### Summary measures of inequality

Recommended visuals for presenting summary measures of inequality include bar graphs, heat maps and Lorenz curves/concentration curves. Bar graphs can be used with any type of summary measure, with the length/area of the bar representing the value of the summary measure. For example, the WHO *State of health inequality: Indonesia* report uses bar graphs to compare the extent of inequality in selected health indicators (Supplementary material 1, Figure A2) [39]. Absolute and relative versions of summary measures of inequality are displayed using bar graphs situated next to each together, providing a more comprehensive representation of inequality.

Heatmaps also have wide applicability for presenting any type of summary measure or combination of summary measures. They can be used to present summary measures that capture multiple inequality dimensions and/or health indicators and can show the results of multiple different types of summary measures, such as presenting difference values alongside ratio values for a given health indicator-inequality dimension combination. In the WHO *State of inequality: HIV*,* tuberculosis and malaria* report, a heatmap was used to show the extent of inequality in malaria indicators in Togo (Supplementary material 1, Figure A3) [40]. It included five dimensions of inequality, and categorized inequality as high, moderate and low, indicating directionality for cases of high and moderate inequality.

Lorenz curves/concentration curves are applicable for illustrating certain summary measures of inequality, such as Gini index or concentration index. In *Health inequality monitoring: harnessing data to advance health equity*, concentration curves visualize the calculation of disproportionality measures for maternal and child health indicators by economic status in Indonesia (Supplementary material 1, Figure A4) [3].

### Scenario 2: change over time in a single setting

Reporting data across multiple time points shows how inequalities have changed over time. Trends of improving, worsening or stagnant health inequality can provide initial insight into the impact of equity-oriented policies, programmes and practices, and where further evidence is needed. Change over time may be visualized using disaggregated data or summary measures, though the presentation of disaggregated data across many subgroups and time points can be a lot of information for the reader to take in. Therefore, summary measures tend to be particularly useful to convey more complex trends.

#### Disaggregated data

Recommended visuals for presenting change over time using disaggregated data include equiplots, bar graphs and line graphs. Equiplots showing the latest status of inequality can be expanded by adding rows (or columns, if oriented vertically) corresponding to each time point. Equiplots can be an efficient way to display a lot of data together in one visual. Equiplots are used in the WHO *State of inequality: childhood immunization* report to show country-level data for childhood immunization indicators, disaggregated by household economic status, mother’s education, place of residence and sex (see, for example, Supplementary material 1, Figure A5) [41]. Disaggregated data from up to five distinct time points are shown for each of the 23 priority study countries.

Bar graphs can be designed in different ways to show disaggregated data across multiple time points. Depending on the desired messaging, bars may be arranged according to the time point (e.g. showing all subgroup data together per each year). This arrangement emphasizes how differences between subgroups have changed from one time point to another. Another approach is to arrange the bars according to the subgroup (e.g. showing data for all available years, per subgroup). This arrangement emphasizes how the level of the health within each subgroup has changed. Confidence intervals can be added to bars to facilitate accurate comparisons across time periods and determine whether differences between bars are statistically meaningful.

Line graphs are also appropriately used to show disaggregated data across multiple time points. Data are plotted with time on the horizontal axis and the health indicator on the vertical axis. Subgroup data are connected using lines (one line per subgroup) that show how the situation within the subgroup has changed over time.

#### Summary measures of inequality

Bar graphs, line graphs and Sankey diagrams are recommended for visualizing change over time using summary measures. Because summary measures convey inequality more efficiently than disaggregated data, bar and line graphs often contain information about multiple indicators or dimensions of inequality. Bars and lines should be clearly labelled on the graph or in an accompanying legend. Sankey diagrams are effectively used if the desired messaging relates to general trends, for example, showing the number of health indicators with high, medium or low inequality, based on a summary measure, at different time points.

### Scenario 3: assessing inequality across multiple settings

Building on scenarios 1 and 2, this scenario assesses inequality across multiple settings. It may involve benchmarking, whereby the level of inequality is compared across similar areas or populations to get a sense of how one area or population performs in relation to others [3]. It may also involve pooling data across settings for a broader overview of the global state of inequality. Reporting may entail presenting disaggregated data to show the latest status of inequality or change over time across settings. It may also entail presenting summary measures of inequality to show latest status or change over time. An additional possibility is the use of visuals to report the setting averages alongside inequality data.

#### Disaggregated data

Recommended visuals for presenting disaggregated data for assessing inequality across multiple settings (either latest status or change over time) include equiplots, box plots, strip plots or violin plots. Equiplots are an efficient way to present disaggregated data for multiple settings. When oriented horizontally, the setting names can be displayed more legibly. If applicable, a setting of interest can be highlighted, allowing for benchmarking.

Box plots, strip plots and violin plots all facilitate comparisons of median subgroup values across settings, as well as the distribution and range of values across subgroups. For these graphs, data are shown using one plot per subgroup at each time point. In strip plots, individual settings of interest can be highlighted for benchmarking. In an analysis of DTP3 (three doses of diphtheria–tetanus–pertussis vaccine) immunization coverage among one-year-olds in 51 low- and middle-income countries, a box plot was used to illustrate the median levels of coverage across five wealth quintiles (Supplementary material 1, Figure A6) [42]. It showed that overall coverage was highest in the wealthiest quintile, and that the range of coverage values was widest among the poorest quintile.

#### Summary measures of inequality, latest status

Recommended visuals for presenting summary measures for the latest status of inequality across multiple settings include bar graphs, Lorenz curves/concentration curves, box plots, strip plots, violin plots, choropleth maps and heatmaps. The use of a bar graph enables each setting to be clearly labelled and compared with other settings. Confidence intervals can also be displayed to show the precision of estimates. The WHO *Explorations of inequality: childhood immunization* report used bar graphs and concentration curves to illustrate economic- and education-related inequality in DTP3 immunization coverage, calculated using concentration index, across 10 priority countries [43]. While bar graphs allow for the clear depiction of the extent of inequality in each setting (one bar for each of the 10 countries) (Supplementary material 1, Figure A7), the concentration curve visual, with 10 curves plotted for the 10 countries, effectively conveys outliers (Supplementary material 1, Figure A8).

Box plots, strip plots and violin plots convey information about the range of within-setting inequality across settings, as expressed by the summary measure. For example, in the WHO *State of inequality: HIV*,* tuberculosis and malaria* report, strip plots show summary measure values across countries for key disease indicators [40]. In supplementary material 1, Figure A9, the difference (based on the richest and poorest economic status quintiles) in study countries is shown for key disease indicators and colour coding is used to enhance the interpretation of the strip plots, indicating countries with high, moderate and low/no inequality, favouring either the richest or the poorest.

Choropleth maps can be a compelling way to visualize summary measures to compare inequalities across settings. For example, it may present the extent of urban-rural inequality for each subnational region within a country. In this way, the map would capture two dimensions of inequality – place of residence and subnational region.

A heatmap can used to present summary measures of inequality across multiple settings and may incorporate several dimensions of inequality and health indicators.

#### Summary measures of inequality, change over time

We recommend bar graphs, line graphs, box plots, strip plots and violin plots for reporting summary measures of inequality to assess change in inequality over time across multiple settings. Bar graphs can use multiple bars per setting to show the extent of inequality at each time point. They tend to be suitable for data over a limited number of time points (to avoid becoming cluttered and difficult to interpret) and are appropriate for situations where all settings have data from common time points. Depending on the arrangement of the data within the graph, bar colour may distinguish the time points or settings.

If there are many time points, or if the time points vary by setting, line graphs tend to be a good choice. For example, line graphs were used to illustrate education-related inequalities in beliefs and behaviours pertaining to COVID-19 interventions over the period of June-December, 2021 (Supplementary material 1, Figure A10) [44]. They show the extent of inequality, calculated using the slope index of inequality, for each month, and use shading to represent the interquartile range.

In this application, two or more box plots (or strip plots or violin plots) can be used to show the distribution of the summary measure across multiple time point – that is, one plot per time point. Violin plots can be layered to facilitate comparisons over time.

#### Setting averages and summary measures of inequality

For visuals reporting setting averages alongside summary measures of inequality for multiple settings, we recommend scatter plots or arrow charts (for change over time). Scatterplots presenting these two variables enable benchmarking across each variable and can help readers identify clusters of settings with similar patterns (for instance, according to the four quadrants). Scatterplots were used in the *State of inequality: reproductive*,* maternal*,* newborn and child health* report, for example, to showcase difference alongside national average (Supplementary material 1, Figure A11) [45]. The median values on both axes were indicated using dotted lines, creating quadrants to showing the settings that were above and below the medians according to the two variables. In the same report, scatterplots were also used to show the change in national average alongside change in inequality over time (Supplementary material 1, Figure A12) [45]. In these scatterplots, the four quadrants contain countries that demonstrated the most desirable, least desirable and mixed changes over time.

Arrow charts are a good choice for presenting setting averages and summary measures for multiple settings at two time points. Using one arrow per setting, they demonstrate how the association between the two variables changes. Johns et al. (2024) used an arrow chart to show absolute economic-related inequality in maternal tetanus immunization coverage and average coverage level, before pregnancy and at birth (Supplementary material 1, Figure A13) [46]. Arrows represented different country income groupings.

### Scenario 4: impact of eliminating inequality

Presenting data about the impact of eliminating inequality can strengthen calls for action in health inequality reporting, especially when the audience includes policymakers. These data can be derived from the summary measures population attributable risk (PAR) and population attributable fraction (PAF), which convey the improvement in the health of the total population that would be achieved if all subgroups had the same level of health indicator as the selected reference group.

Data visuals recommended for reporting the impact of eliminating inequality include stacked bar graphs, bullet graphs and Sankey diagrams. Stacked bar and bullet graphs contain information about the current situation (the current setting average), potential improvement (the PAR or PAF value) and the potential future situation (the setting average in the case of eliminating inequality). Stacked bar graphs use the two portions of the bar to show this: the lower section shows the current average, the upper section shows the potential improvement, and the total length of the bar represents the potential setting average if there was no inequality. In a bullet graph, the bar shows the current setting average, and the line shows the potential future situation if there was no inequality; the gap between the bar and the line indicates the potential increase (or decrease) in the setting average. While bullet graphs can be used with any health indicator, stacked bar graphs are limited to reporting of situations when the setting average increases when inequality is eliminated. In most cases, this means health indicators that have a positive relationship with health, such that higher values are generally regarded as better [3].

The *State of inequality: reproductive*,* maternal*,* newborn and child health* report uses stacked bar graphs to compare the potential for improvement by eliminating within-country economic-related inequality in selected health intervention indicators in Egypt versus Niger (Supplementary material 1, Figure A14) [45]. In this case, increases in national averages were evident for all indicators, making stacked bar graph an appropriate choice.

In the *State of inequality: HIV*,* tuberculosis and malaria* report, bullet graphs were used to show the potential improvement in national average by eliminating economic-related inequality across selected HIV, tuberculosis and malaria indicators (Supplementary material 1, Figure A15) [40]. For one indicator related to catastrophic health costs, the elimination of inequality resulted in a decrease in national average, whereas other indicators demonstrated increases in national averages. Thus, a bullet graph was suitable.

Sankey diagrams may be used to convey general potential trends, given the potential elimination of inequality. For example, a Sankey diagram in the WHO *World health statistics 2025* report shows the current distribution of countries reporting low, medium, high or very high economic-related inequality in DTP3 coverage (left nodes) (Supplementary material 1, Figure A16) [47]. The nodes on the right of the graph indicate the potential distribution of countries across inequality levels if economic-related inequality were eliminated.

## Discussion

A wide range of data visual types are used in health inequality reporting, with different suitability depending on the nature of the data being reported, the desired messaging and the reporting context. Bar graphs, line graphs and equiplots are among the most adaptable data visuals for different scenarios of reporting. Equiplots are especially suitable for reporting disaggregated data and line graphs are highly appropriate for reporting trends over time. A key advantage of bar graphs, line graphs and equiplots is their use of the underlying “L-shaped” or “x-y coordinate” graph schema, which tends to be familiar to many audiences [20, 48]. Sankey diagrams, heatmaps and choropleth maps can be effectively used for reporting purposes that group data according to defined ranges or thresholds, and where presenting data points with high precision is not a priority. Other visual types, namely Lorenz curves, concentration curves, scatterplots, arrow charts, box plots, strip plots and violin plots, have more specialized applications.

This paper presents four scenarios for reporting health inequality data, which demonstrate several technical considerations that should be taken into account when selecting data visuals. These include the type of data measure being presented (e.g. disaggregated data versus summary measures of inequality), the number of settings represented in the visual and the inclusion of data for a single time point versus multiple time points. Although these each pose distinct considerations, taken together, they suggest that the volume of data points included in a data visual is a pertinent factor. Practically, different data visuals may pose trade-offs between data precision and quantity. For instance, bar graphs and equiplots allow data to be presented more precisely, but there are limitations in the amount of data that can be reasonably included in a bar graph or equiplot without appearing cluttered and hindering interpretation. Sankey diagrams, box plots, strip plots and violin plots can accommodate a larger quantity of data points, though generally with lower precision.

The purpose of reporting, which may be determined prior to undertaking the analysis, also weighs into the selection and design of data visuals [3]. The answers to questions such as “why is reporting being done?” and “who is the report meant to reach?” can help to formulate key messages and ensure that the data visuals effectively highlight these messages in a manner that is meaningful to the intended audience. For example, the communication of confidence intervals within graphs may be appropriate for technical audiences, which affects the choice of graph (e.g. the use of bar and line graphs instead of equiplots and strip plots). Migita et al. (2023) documented their experiences preparing data visuals to highlight racial inequalities in a healthcare setting [49]. They describe a fit-for-purpose data dashboard grounded in simplicity that enabled data to be quickly conveyed to clinical teams, who could then take action in a timely fashion. A review by Chau et al. (2024) highlighted the emerging potential of community-centered methods to create health-related data visuals and give communities an “active voice in data storytelling” that is geared towards them [50]. It is less, clear, however, what implication this has for what visualization types can be used, adapted, or may need to be added. This is an area for further development.

This paper intends to convey key considerations and recommendations for visualizing health inequality data. We have raised certain conventions for the preparation of the data visuals featured in this paper – some general best practices for accurately presenting inequality data in graphs are summarized in Box 2 – though there are contextual considerations that may be pertinent to account for. For instance, the selection of colours should take into consideration the cultural context – red, for example, may signify danger or warning in some cultures, and good luck in other cultures – and colour palettes should aim to be colour-blind-friendly. Audiences with diverse disciplinary backgrounds may have different data literacy reference points. A data visualization literacy framework by Börner et al. (2019) provides common definitions and core concepts to describe aspects of data visuals, underscoring the utility of a more systematic approach to constructing and interpreting visuals [48].

**Box 2:** Best practices for presenting inequality data using graphs

If the graph presents data across multiple time periods, ensure that time is represented consistently in axis spacing. This is especially relevant when data are available at irregular intervals.
For multiplicative measures such as ratio and relative index of inequality, use a logarithmic scale for the corresponding graph axis, such that results are displayed in accordance with the extent of inequality they represent.
Avoid elongating or inverting axes, as this can distort and misrepresent data. Avoid truncating axes on graphs that present data using the height or length of objects to encode values, such as bar graphs.
Include clear and comprehensive captions to ensure that graphs can be interpreted if reproduced out of their original context.
For more information about technical considerations for reporting health inequality data, see *Health inequality monitoring: harnessing data to advance health equity* [3].

Four illustrative reporting scenarios were highlighted, however, these are not comprehensive of all reporting scenarios or inequality data. For example, while the first three scenarios highlighted in this paper focus on the reporting of disaggregated data and summary measures of health inequality, there are other types of inequality data that may be reported, such as odds ratios produced from regression analysis (which are often presented using bar charts, as in the *Explorations of inequality: childhood immunization* report [43]).

Given the many possible nuances of data analyses and the complexities of communicating data, data visual types and designs not covered in this paper may prove effective for different audiences and key messages. We acknowledge that our approach of reviewing articles in one journal (IJEqH) to verify our base list of data visual types introduces a potential selection bias, as the inclusion of other journals may have led us to include other visual types. Further empirical research is warranted to study the effectiveness of a wider selection of health inequality data visuals in different contexts.

Through a variety of examples, we sought to illustrate some of the applications of common data visual types across diverse health topics. Many of the featured scenarios indicate several possible data visual types that may be appropriate. Because a “switch cost” has been observed when readers move from one graph type to another [20], it is advisable to limit the use of different data visuals within a common reporting output [3]. To promote the accessibility of information within data visuals, colours, labelling and annotations should be used judiciously [17, 51].

## Conclusions

Graphs and maps – either static or interactive – are widely used in health data communication. Data visuals have the potential to streamline the analysis, interpretation and uptake of health inequality data as evidence to advance health equity. This paper has synthesized considerations related to the effectiveness of common data visuals used for reporting health inequality data and suggested visuals that may be most appropriate for different health inequality reporting scenarios. Readers are encouraged to consider the reporting purpose and target audience, the analytical approach, the intended messaging and the characteristics of the underlying data when selecting and designing graphs and maps to present health inequality data.

## Supplementary Information

Below is the link to the electronic supplementary material.

Supplementary Material 1

## Data Availability

No datasets were generated or analyzed during the current study.

## Footnotes

1 When presenting data for multiple settings, the median value is often reported rather than an average. This represents the middle value of the dataset and is less influenced by outliers (in the case of unweighted average) or disparate population sizes (in the case of weighted average).

## Abbreviations

DTP3: Three doses of diphtheria–tetanus–pertussis vaccine
HEAT: Health Equity Assessment Toolkit
IJEqH: International Journal for Equity in Health
PAF: Population attributable fraction
PAR: Population attributable risk
WHO: World Health Organization
