# Supplementary material for: Visualizing health inequality data: guidance for selecting and designing graphs and maps
Source: Int J Equity Health. 2025 Dec 11;24:343. doi: 10.1186/s12939-025-02667-0 (PMC12699797; doi:10.1186/s12939-025-02667-0)
Supplement: Supplementary file 1 — Supplementary Material 1 [file 12939_2025_2667_MOESM1_ESM.docx]

# Additional file 1

The figures in the file serve as examples of how different data visual types have been applied in publications reporting on the state of inequality. Please refer to the original sources for further information about the underlying data, analysis and reporting context.

## Scenario 1: Latest status of inequality in a single setting

**Figure A1. Example of choropleth map showing subnational regional inequality in access to improved drinking water across 34 regions in Indonesia, 2015**


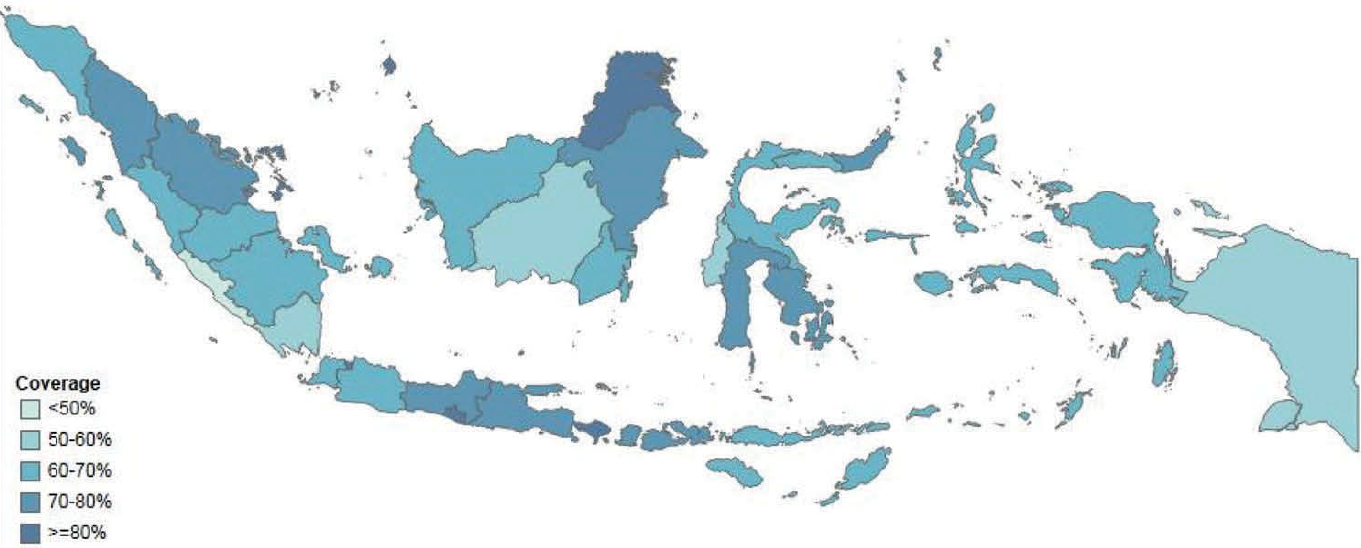


Reproduced from Afifah et al (1)

**Figure A2. Example of bar graph comparing the extent of subnational regional inequality in public health development indices, calculated as mean difference from mean and index of disparity in Indonesia****, 2011-2013**


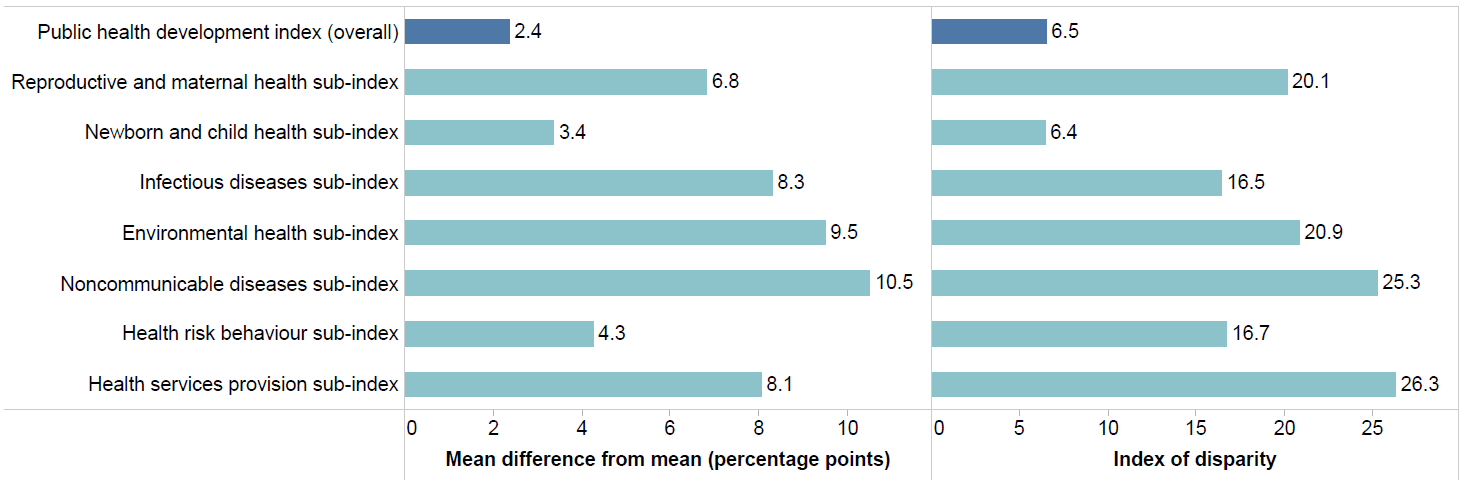


Reproduced from World Health Organization (2)

**Figure A3. Example of a heatmap showing the extent of inequality in malaria indicators in Togo, 2017**


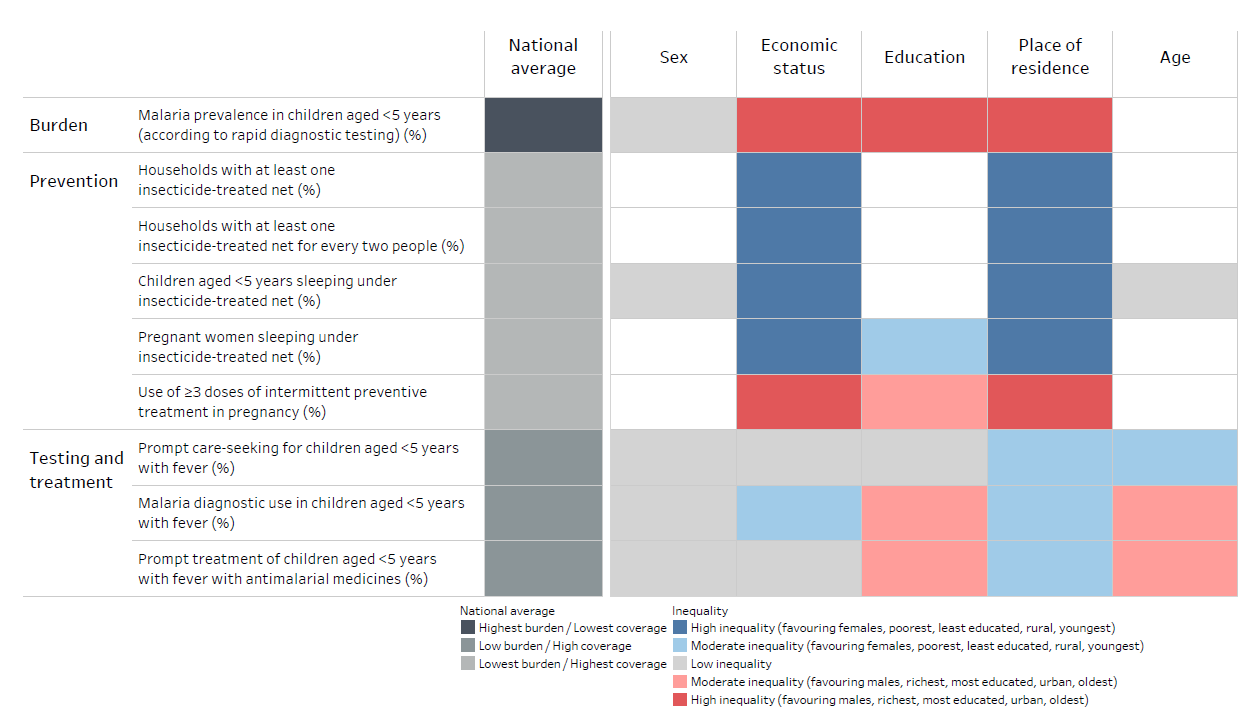
Reproduced from World Health Organization (3)

**Figure A4. Example of concentration curves visualizing the calculation of disproportionality measures for maternal and child health indicators, by economic status, in Indonesia, 2007**

**
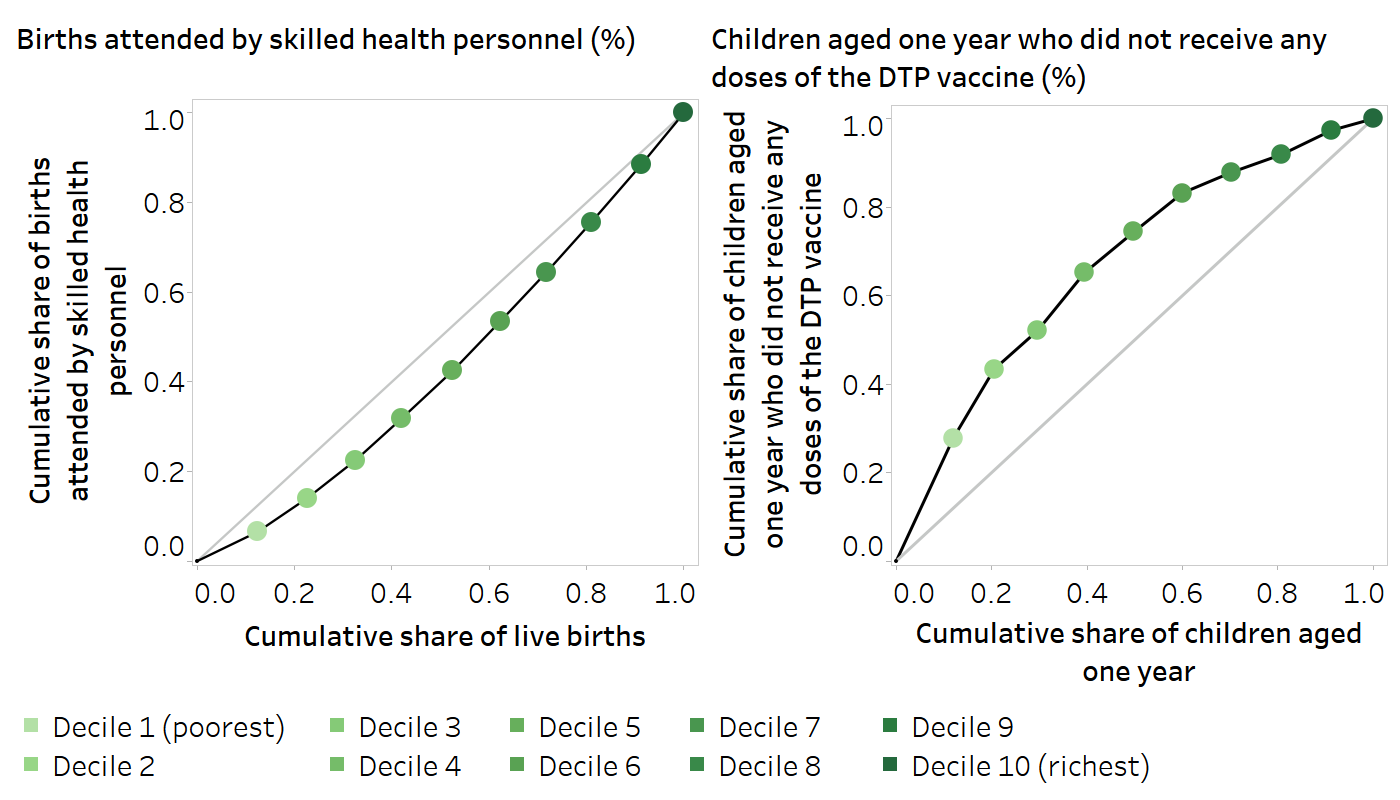
**

DTP: diphtheria, tetanus toxoid and pertussis

Reproduced from World Health Organization (4)

## Scenario 2: Change over time in a single setting

**Figure A5. Example of equiplots showing country-level data for childhood immunization indicators, disaggregated by household economic status, mother’s education, place of residence and sex in Ethiopia, 2000, 2005, 2011**


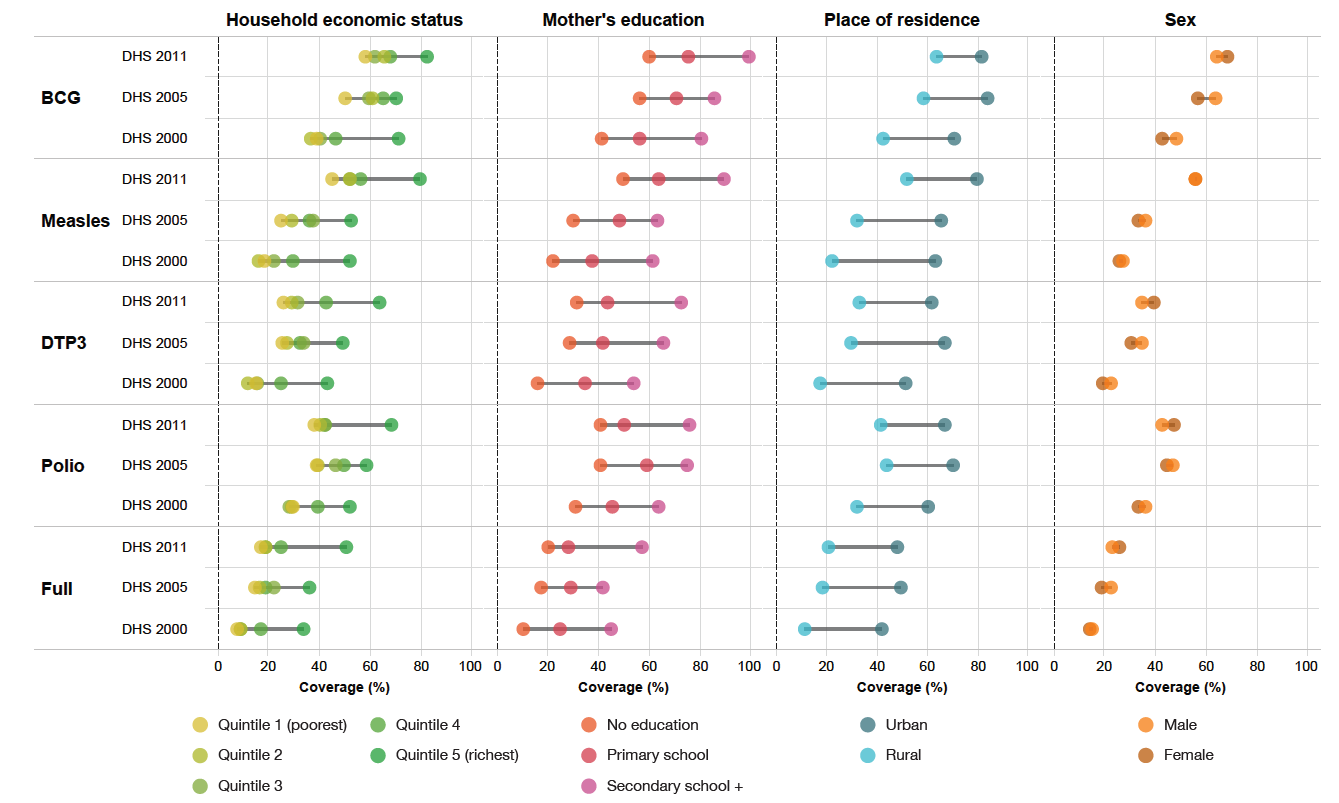


BCG: bacille Calmette-Guérin; DHS: Demographic and Health Survey; DTP3: three doses of diphtheria, tetanus toxoid and pertussis vaccine

Reproduced from World Health Organization (5)

## Scenario 3: Assessing inequality across multiple settings

**Figure A6. Example of box plot reporting DTP3 immunization coverage among one-year-olds, by economic status quintiles, in 51 low- and middle-income countries, 2010-2013**


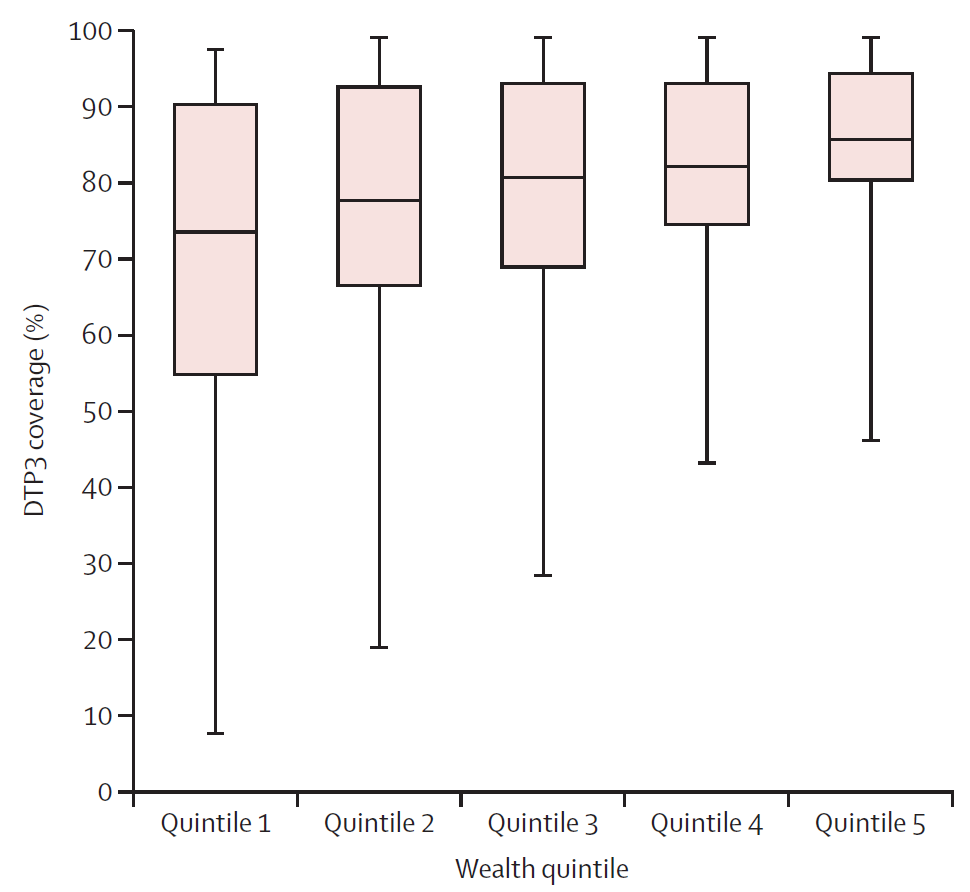


DTP3: three doses of diphtheria, tetanus toxoid and pertussis vaccine

Reproduced from Hosseinpoor et al (6)

**Figure A7. Example of bar graph to illustrate economic-related inequality in DTP3 immunization coverage, calculated using concentration index, across 10 priority countries, 2012-2016**


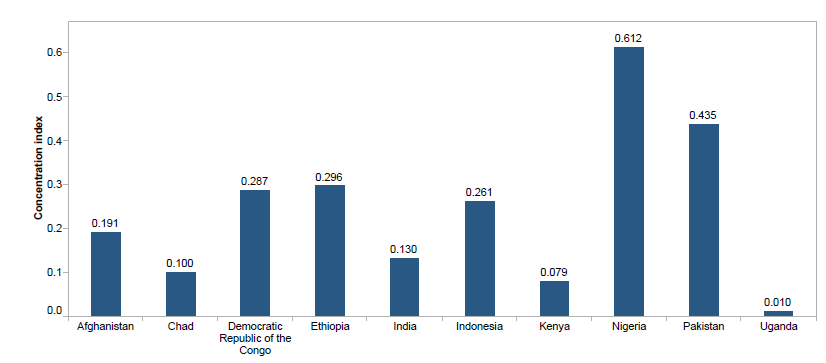


DTP3: three doses of diphtheria, tetanus toxoid and pertussis vaccine

Reproduced from World Health Organization (7)

**Figure A8. Example of concentration curve to illustrate economic-related inequality in DTP3 immunization coverage across 10 priority countries, 2012-2016**


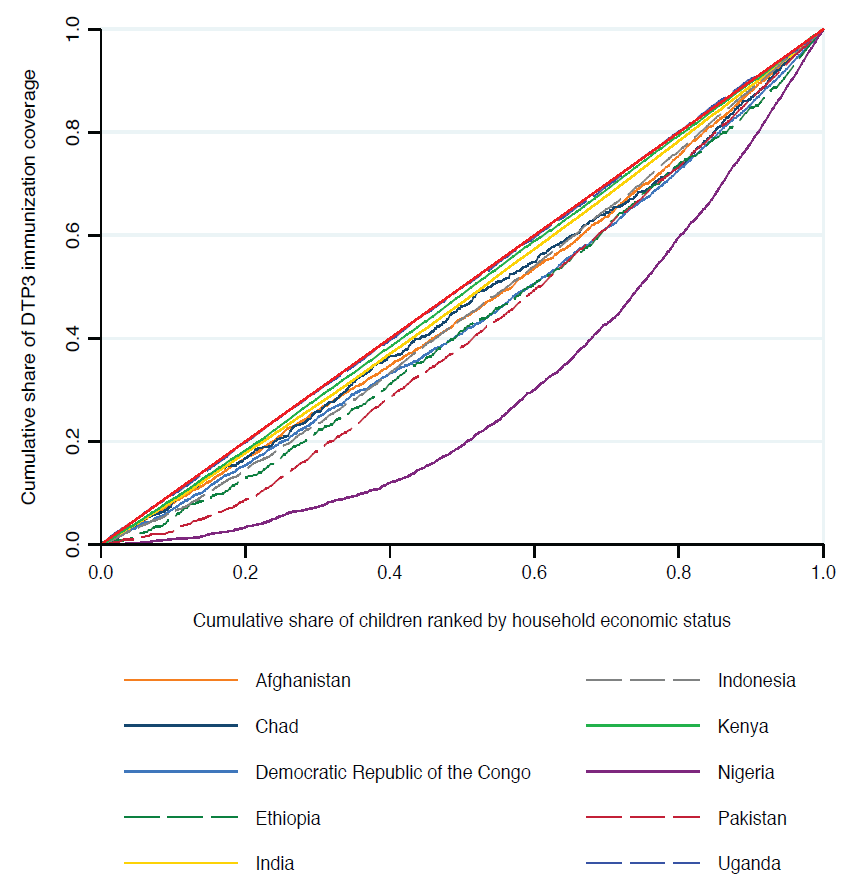


DTP3: three doses of diphtheria, tetanus toxoid and pertussis vaccine

The red diagonal line represents the line of equality.

Reproduced from World Health Organization (7)

**Figure A9. Example of strip plots showing difference (based on richest and poorest economic status quintiles) in study countries for key HIV, tuberculosis and malaria indicators, 2011-2020**


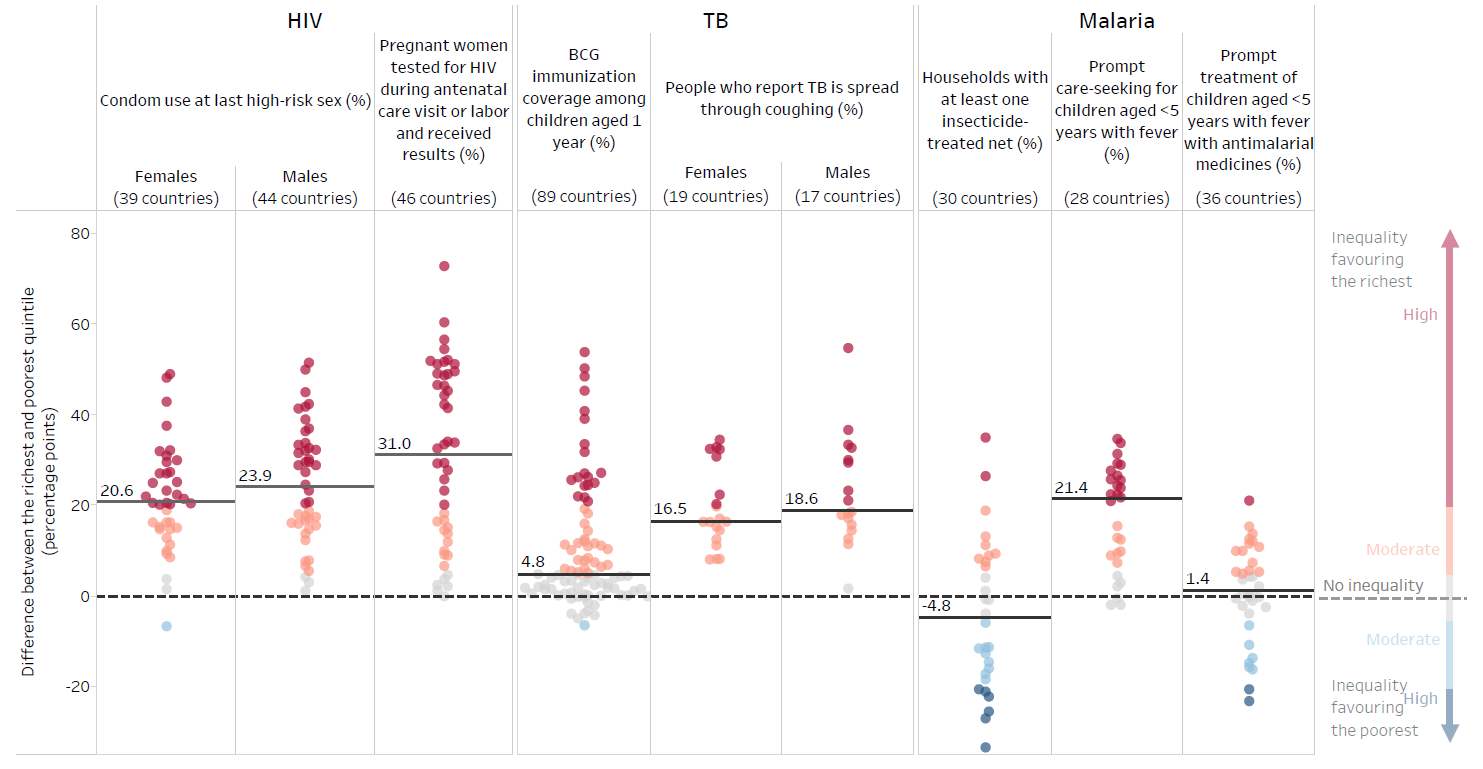


BCG: bacille Calmette-Guérin; TB: tuberculosis

Circles indicate countries. Solid horizontal lines indicate the median value across all countries with data.

Reproduced from World Health Organization (3)

**Figure A10. Example of line graphs illustrating education-related inequalities in beliefs and behaviours pertaining to COVID-19 interventions, June-December, 2021**


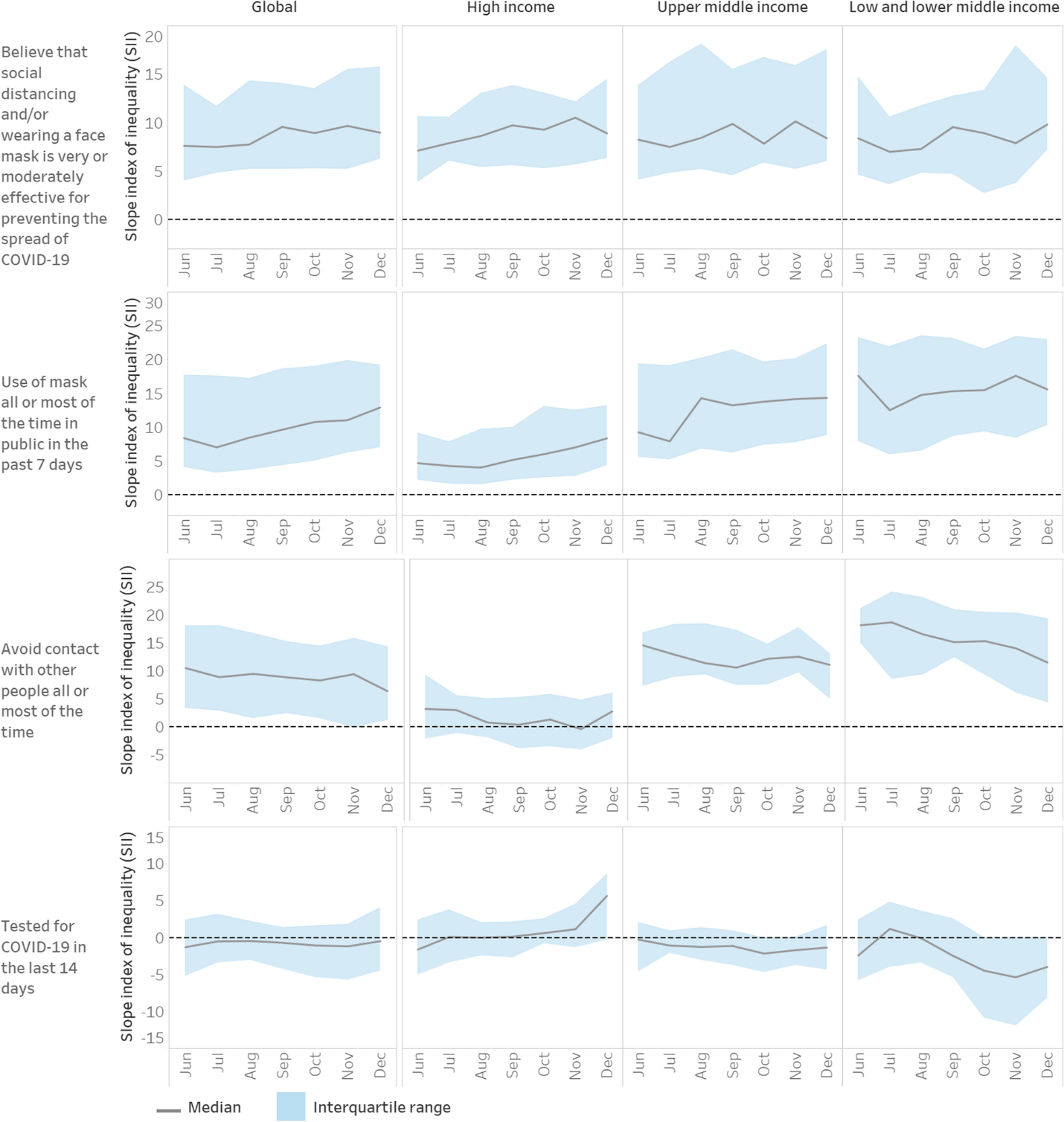


Reproduced from Kirkby et al (8)

**Figure A11. Example of a scatterplot showing economic-related difference and national average in births attended by skilled health personnel, 83 low- and middle-income countries, 2005-2013**


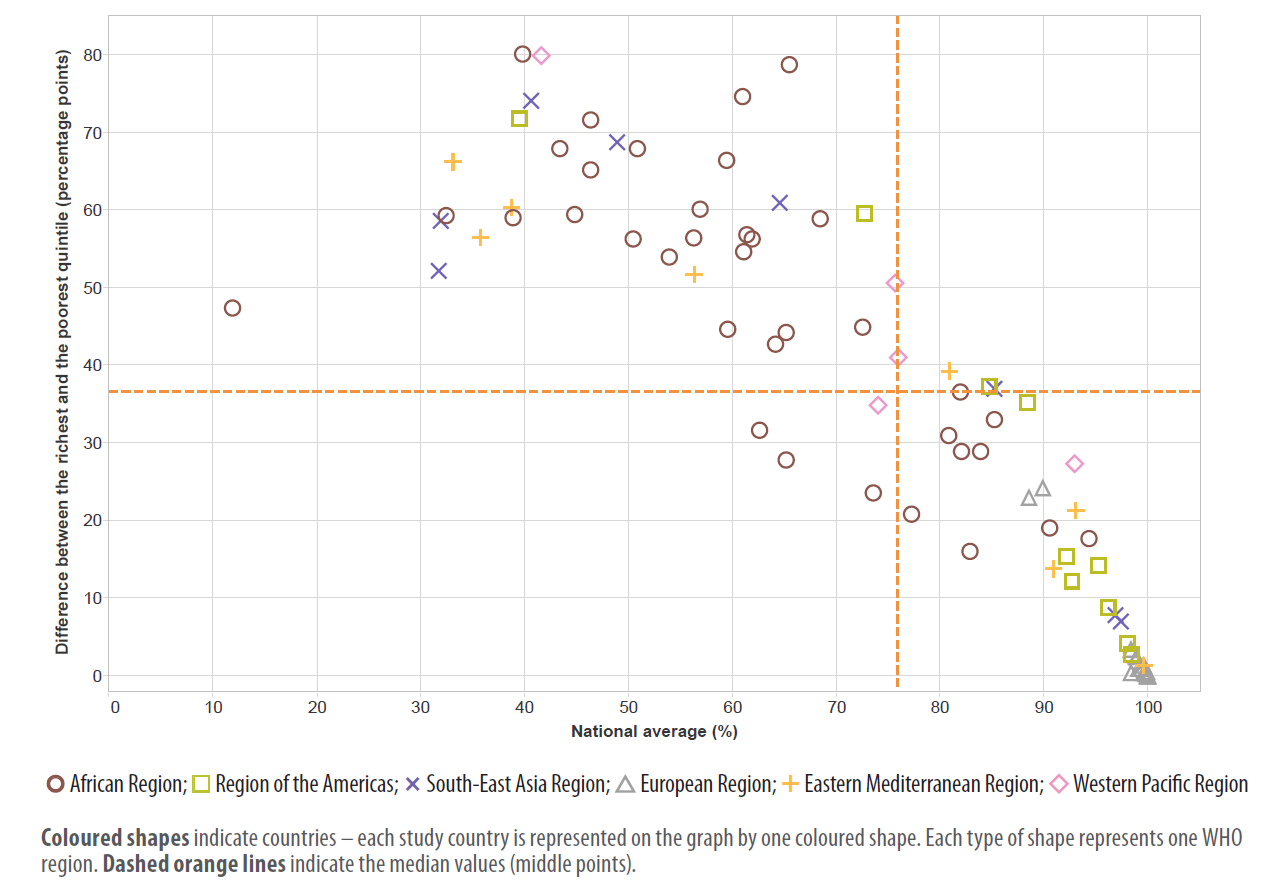


Coloured shapes indicate countries – each study country is represented on the graph by one coloured shape. Each type of shape represents one WHO region. Dashed orange lines indicate the median values (middle points).

Reproduced from World Health Organization (9)

**Figure A12. Example of a scatterplot showing annual absolute change in place of residence inequality and annual absolute change in national average in care seeking for pneumonia symptoms, 33 low- and middle-income countries, 1995-2004 and 2005-2013**


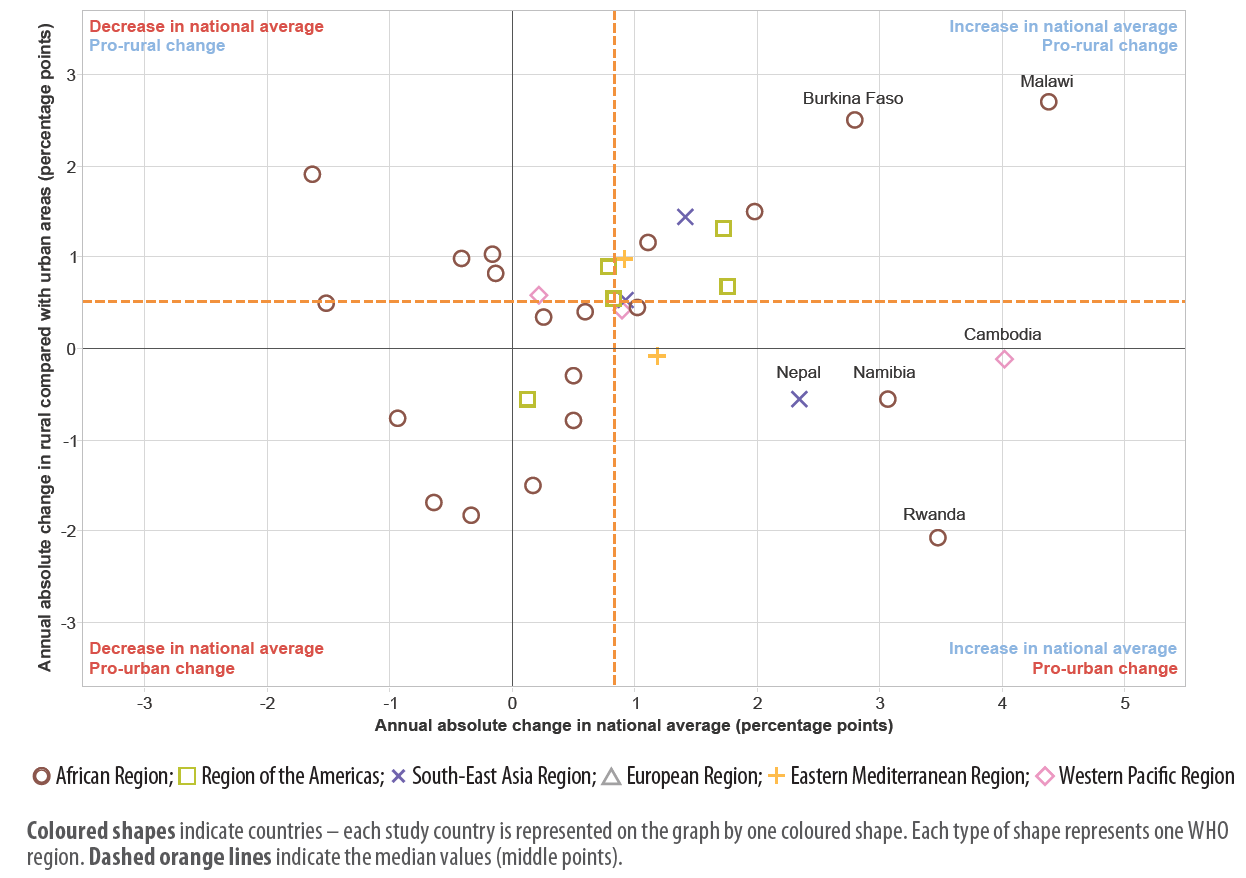


Coloured shapes indicate countries – each study country is represented on the graph by one coloured shape. Each type of shape represents one WHO region. Dashed orange lines indicate the median values (middle points).

Reproduced from World Health Organization (9)

**Figure A13. Example of arrow chart showing economic-related inequality in maternal tetanus immunization coverage and average coverage level, before pregnancy and at birth**


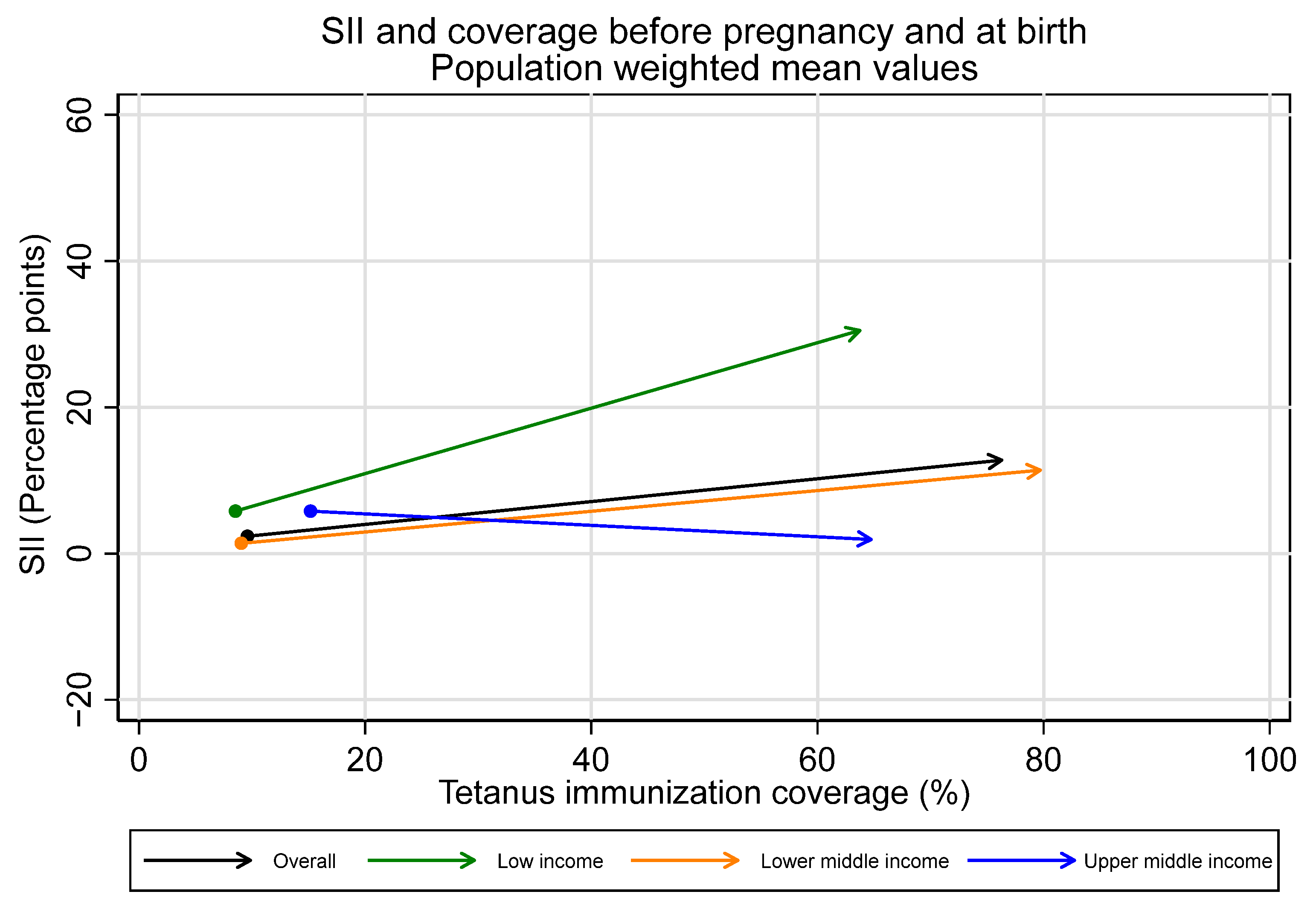


SII: slope index of inequality

Dot represents coverage level and SII in tetanus immunization coverage before pregnancy; arrowhead represents coverage and SII at birth.

Reproduced from Johns et al (10)

## Scenario 4: Impact of eliminating inequality

**Figure A14. Example of a stacked bar chart showing potential for improvement in RMNCH intervention coverage by eliminating within-country economic-related inequality in Egypt (2008) and Niger (2012)**


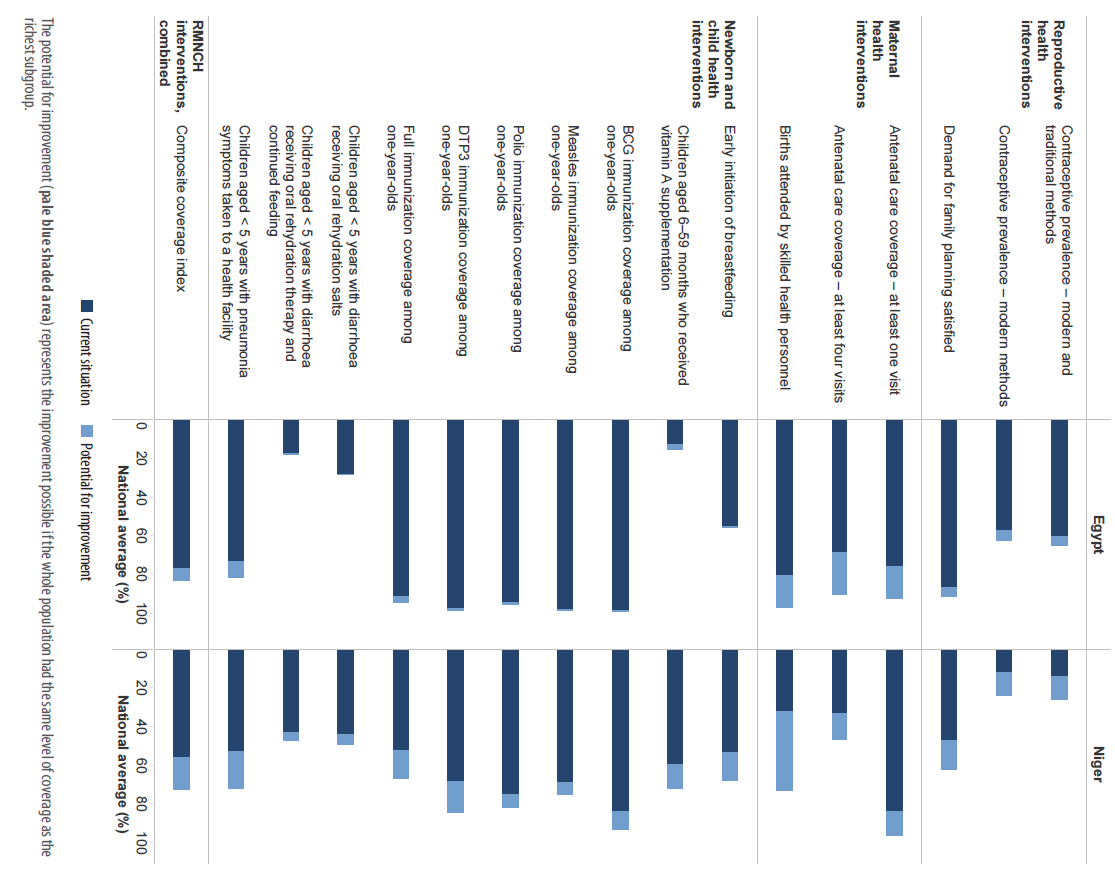


BCG: bacille Calmette-Guérin; DTP3: three doses of diphtheria, tetanus toxoid and pertussis vaccine

The potential for improvement (pale blue shaded area) represents the improvement possible if the whole population had the same level of coverage as the richest subgroup.

Reproduced from World Health Organization (9)

**Figure A15. Example of bullet graphs showing the potential improvement in national average by eliminating economic-related inequality across HIV, tuberculosis and malaria indicators, 2010-2020**


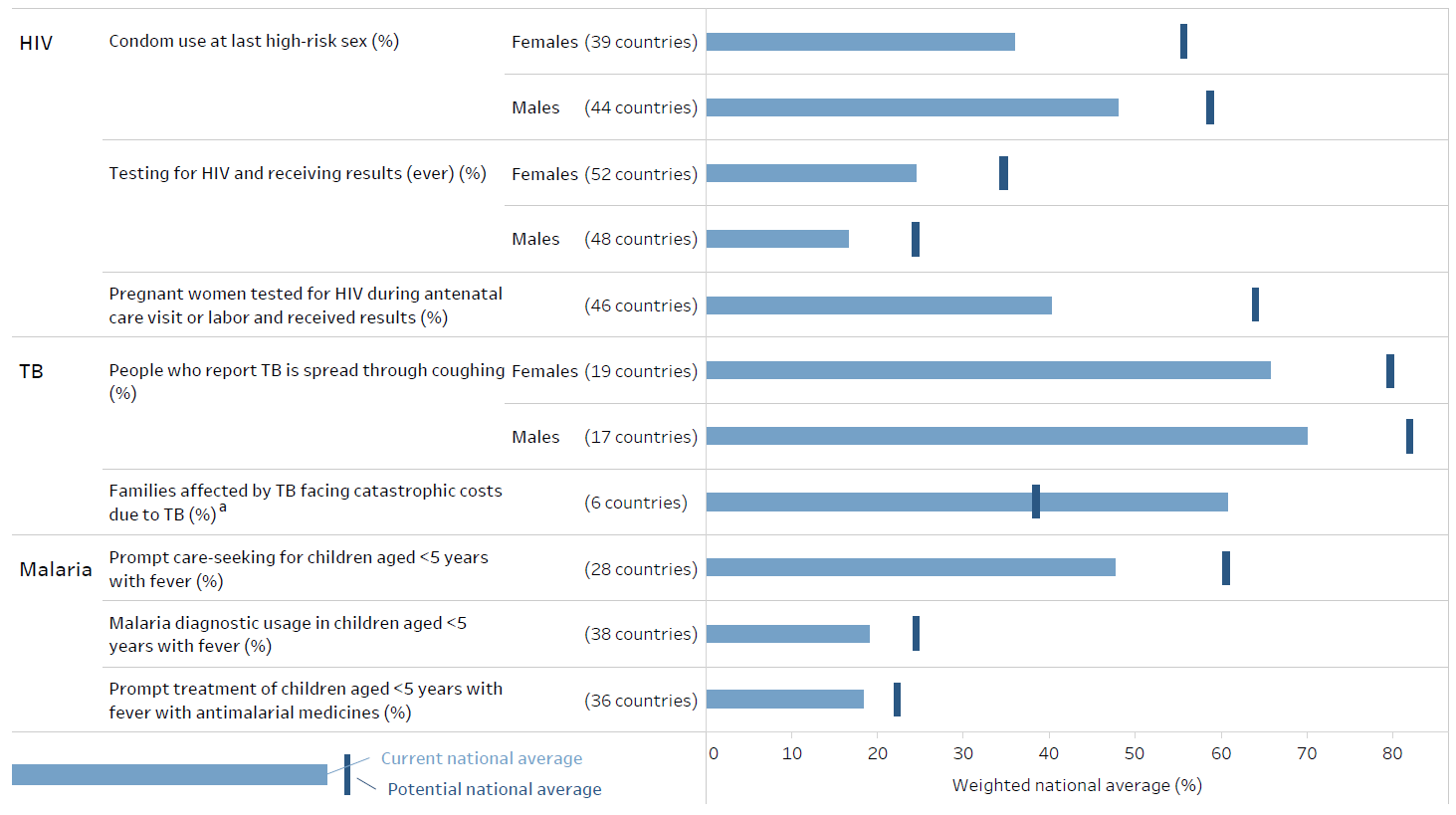
TB: tuberculosis

Reproduced from World Health Organization (3)

**Figure A16. Example of Sankey diagram showing the potential improvement in national average by eliminating economic-related inequality in DTP3 coverage, 88 countries, 2014–2023**


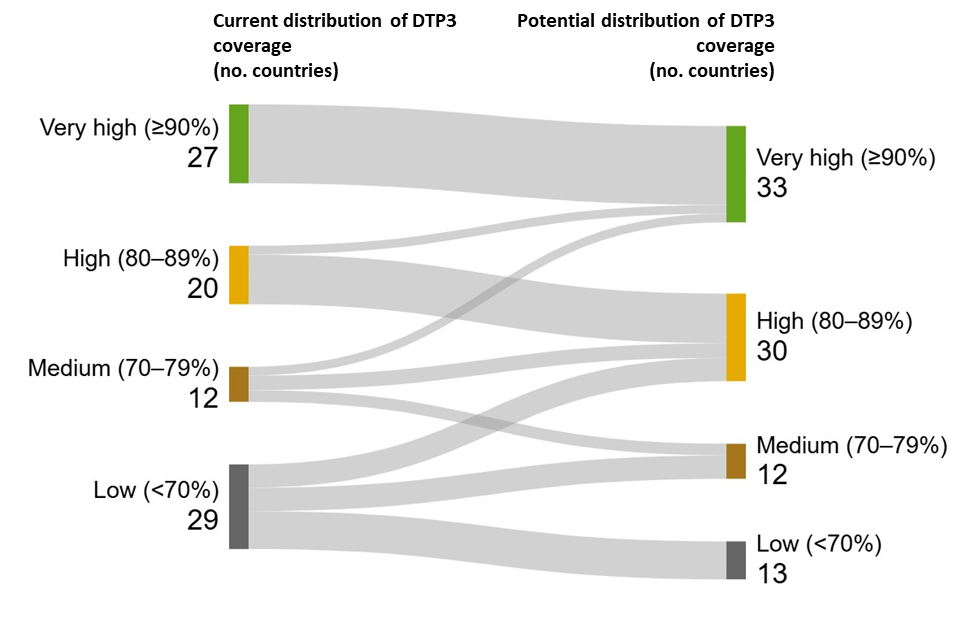


DTP3: three doses of diphtheria, tetanus toxoid and pertussis vaccine

Reproduced from World Health Organization (11)

## References

1. Afifah T, Nuryetty MT, Cahyorini, Musadad DA, Schlotheuber A, Bergen N, et al. Subnational regional inequality in access to improved drinking water and sanitation in Indonesia: results from the 2015 Indonesian National Socioeconomic Survey (SUSENAS). Global health action. 2018;11(sup1):31–40.

2. World Health Organization. State of health inequality: Indonesia [Internet]. Geneva: World Health Organization; 2017. Available from: https://iris.who.int/handle/10665/259685

3. World Health Organization. State of inequality: HIV, tuberculosis and malaria [Internet]. Geneva: World Health Organization; 2021. Available from: https://iris.who.int/handle/10665/350198

4. World Health Organization. Health inequality monitoring: harnessing data to advance health equity [Internet]. Geneva: World Health Organization; 2024. Available from: https://iris.who.int/handle/10665/379703

5. World Health Organization. State of inequality: childhood immunization [Internet]. Geneva: World Health Organization; 2016. Available from: https://iris.who.int/handle/10665/252541

6. Hosseinpoor AR, Bergen N, Schlotheuber A, Gacic-Dobo M, Hansen PM, Senouci K, et al. State of inequality in diphtheria-tetanus-pertussis immunisation coverage in low-income and middle-income countries: a multicountry study of household health surveys. Lancet Glob Health. 2016;4(9):e617-626.

7. World Health Organization. Explorations of inequality: childhood immunization [Internet]. Geneva: World Health Organization; 2018. Available from: https://iris.who.int/handle/10665/272864

8. Kirkby K, Bergen N, Vidal Fuertes C, Schlotheuber A, Hosseinpoor AR. Education-related inequalities in beliefs and behaviors pertaining to COVID-19 non-pharmaceutical interventions. Int J Equity Health. 2022;21(S3):158.

9. World Health Organization. State of inequality: reproductive, maternal, newborn and child health [Internet]. Geneva: World Health Organization; 2015. Available from: https://iris.who.int/handle/10665/164590

10. Johns NE, Blumenberg C, Kirkby K, Allorant A, Costa FDS, Danovaro-Holliday MC, et al. Comparison of wealth-related inequality in tetanus vaccination coverage before and during pregnancy: a cross-sectional analysis of 72 low- and middle-income countries. Vaccines. 2024;12(4):431.

11. World Health Organization. World health statistics 2025: monitoring health for the SDGs, Sustainable Development Goals [Internet]. Geneva: World Health Organization; 2025. Available from: https://iris.who.int/handle/10665/381418
